# MRI Anatomy of *Pseudopus apodus*: A Descriptive Study with High-Field and Low-Field MRI

**DOI:** 10.3390/ani16193007

**Published:** 2026-09-24

**Authors:** María Isabel García-Real, Encarnación Fernández-Valle, David Castejón-Ferrer, Nerea Moreno, Pilar Pérez-Lloret, Sara Jiménez, Raquel Salgüero-Fernández, Miguel Angel Cabezas, María Ardiaca-García, María José Ruiz-Fernández, Juncal González-Soriano

**Affiliations:** 1 Department of Animal Medicine and Surgery, Faculty of Veterinary, Complutense University, Avenida Puerta de Hierro s/n, 28040 Madrid, Spain; rsalgu01@ucm.es (R.S.-F.); dolorvet@gmail.com (M.A.C.); 2 ICTS Bioimagen Complutense, Complutense University, Paseo de Juan XXIII 1, 28040 Madrid, Spain; evalle@ucm.es (E.F.-V.); dcastejon@ucm.es (D.C.-F.); 3 Department of Cell Biology, Faculty of Biological Sciences, Complutense University, Avenida José Antonio Nováis 12, 28040 Madrid, Spain; nerea@bio.ucm.es; 4 Department Section of Anatomy and Embriology, Faculty of Veterinary, Complutense University, Avenida Puerta de Hierro s/n, 28040 Madrid, Spain; pilper01@ucm.es (P.P.-L.); juncalgs@vet.ucm.es (J.G.-S.); 5 Achucarro Basque Center for Neuroscience, Scientific Park of the University of the Basque Country (UPV/EHU), Leioa, 48940 Bilbao, Spain; sara.jimenez@achucarro.org; 6 Veterios Hospital Veterinario, Arrastaria 23, 28022 Madrid, Spain; 7 Dolorvet Veterinary Anesthesia & Analgesia, 28009 Madrid, Spain; 8 Medivet 24 Horas Los Sauces, Chamberi. Calle de Sta Engracia, 63, Chamberí, 28010 Madrid, Spain; ardiaca.m@outlook.com; 9 DB Diagnóstico por Imagen Veterinario (DIAGERVET S.L.), C/Tordesillas 4, Alcorcón, 28925 Madrid, Spain; mariajoseruizfernandez@gmail.com

**Keywords:** *Pseudopus apodus*, European glass lizard, magnetic resonance imaging, anatomy, reptiles, high-field MRI, low-field MRI

## Abstract

*Pseudopus apodus* is an anguimorph lizard belonging to the order Squamata. Anatomical information on this species remains limited, particularly regarding soft tissues, and has primarily focused on the axial skeleton of both living and fossil specimens. Recently, the anatomy of its coelomic cavity has been described using dissection and multi-detector computed tomography (CT), and its brain has been characterized neuroanatomically by combining ultra-high-resolution magnetic resonance imaging (MRI) with histological analysis using immunofluorescence. In the present study, four specimens (one female and three males) were examined. One male and the female were scanned using a low-field MRI system (0.23 T), whereas all three males were scanned using a high-field MRI system (1.5 T). Both MRI systems enabled the identification of a wide range of hard and soft anatomical structures. Only a few small structures could not be consistently identified with the low-field system, including the thyroid gland, spleen, pancreas, testes, and hemipenes. Despite the lower field strength, small ovarian follicles and the optic nerves were clearly visualized with the low-field MRI system. This study provides the first whole-body MRI anatomical reference for *Pseudopus apodus*, complements previous CT-based anatomical studies, and offers practical information for the interpretation of MRI examinations in reptiles.

## Simple Summary

*Pseudopus apodus* is a large legless lizard belonging to the family *Anguidae*. Although its body plan resembles that of snakes, it retains several characteristic lizard features. Anatomical information on this species remains limited and has mainly focused on the description of skeletal structures, based on both living individuals and, particularly, fossil specimens. The present study describes the normal anatomy of *Pseudopus apodus* using whole-body magnetic resonance imaging (MRI). Images were obtained with a high-field MRI system (1.5 T) and a low-field MRI system (0.23 T). MRI provided excellent soft tissue contrast and was particularly useful for identifying the central nervous system, visceral organs, and gonads. In addition, many hard anatomical structures were adequately visualized. Although visualization of very small structures was generally more limited with the low-field MRI system, ovarian follicles and the optic nerves were clearly identified in the low-field examinations. The aim of this study was to describe the normal whole-body MRI anatomy of *Pseudopus apodus* using high-field and low-field MRI.

## 1. Introduction

The *Pseudopus apodus* is a large legless lizard of the family Anguidae distributed from southeastern Europe to western Asia [1]. Despite its distinctive morphology, anatomical information on this species remains fragmentary and derives mainly from classical descriptions and comparative morphological studies [1,2,3,4,5]. Previous studies have focused on the skull, vertebral column, appendicular skeleton, and pulmonary asymmetry, whereas detailed imaging-based soft tissue descriptions remain limited [2,3,4,5,6].

In exotic medicine, magnetic resonance imaging (MRI) is considered an imaging technique particularly well suited for the evaluation of soft tissues because it provides excellent contrast resolution for the central nervous system, viscera, and reproductive organs [7,8,9,10]. Nevertheless, the high cost of equipment, the lengthy duration of the scanning procedure (particularly with low-field MRI) and the lack of detail encountered in small patients are factors that have limited the more frequent use of this technique in snakes and lizards [11]. Moreover, detailed descriptions of the normal anatomical MRI features of reptiles remain scarce, and most imaging studies have focused on selected body regions [12,13,14,15,16,17,18,19,20,21,22,23,24,25,26].

MRI scanners are classified into three categories: low-field (0.2–0.4 T), medium-field (0.5–1 T), and high-field (>1 T). High-field systems have high costs for purchasing, running and maintenance, but provide higher quality images with shorter acquisition times. In contrast, low-field MRI scanners have a lower cost and easier installation and maintenance but have lower signal-to-noise ratio compared to high-field devices. This limitation leads to lower spatial resolution and longer acquisition times. Therefore, due to the low signal-to-noise ratio, it can be challenging to obtain diagnostic-quality images in small patients [27,28,29].

A recent study has provided a detailed description of anatomical dissection and multi-detector CT features of the non-skeletal structures of *Pseudopus apodus*. The vomeronasal organ with the choanae, tongue, glottis, hyoid bone, esophagus, stomach, small and large intestines, cloaca, liver, gallbladder, kidneys, ovarian follicles, trachea, bronchial bifurcation, lungs, heart, aortic arches, dorsal aorta, sinus venosus, and cranial cava veins were identified in both the anatomic dissection and CT images. However, the thyroid, pancreas, spleen, ureters, urinary bladder, oviducts, testes, hemipenes, pulmonary trunk, and pulmonary arteries were seen in the anatomical dissection but not in the CT studies [30].

The aim of the present study was to describe the normal whole-body anatomy of *Pseudopus apodus* using both high-field and low-field MRI, with emphasis on soft tissue structures, although numerous hard anatomical structures were also documented.

## 2. Materials and Methods

### 2.1. Animals

For the present study, four individuals (one female and three males) of *Pseudopus apodus* with good body condition were used. One male and the female were scanned using a low-field MRI system, whereas all three males were scanned using a high-field MRI system. Specimens were adult and healthy, and they were obtained from commercial pet suppliers. Animals were handled in accordance with the guidelines for animal research set out in the European Community Directive 2010/63/EU [31] and following the recommendations of the European Commission for the protection of animals used for scientific purposes. All procedures were approved by the local ethics committee (O.H. (CEA)-UCM—NP0409032022-2022).

### 2.2. Anesthesia and Positioning

MRI examinations were performed under general anesthesia to minimize motion and stress. The anesthetic protocol consisted of intramuscular (IM) alfaxalone at a dose of 4 mg/kg, which facilitated endotracheal intubation. Isoflurane was subsequently administered and titrated to effect with a fraction of inspired oxygen (FiO_2_) of 0.5. Respiratory rate and CO_2_ levels were monitored continuously throughout the procedure, remaining within physiological ranges for the species. In all cases, immobility was achieved, and spontaneous ventilation was successfully maintained.

### 2.3. MRI Protocol

Imaging was performed using two different MRI systems: a clinical Vantage Elan 1.5 T MRI system (Canon Medical, Tokyo, Japan) equipped with a whole-body radiofrequency coil for transmission and a 16-channel flexible phased-array coils for reception, and a clinical Panorama 0.23 T open MRI system (Philips Medical Systems, Best, The Netherlands) with a volume whole-body radiofrequency coil for transmission and a flexible coil for reception.

Sequence selection was based on preliminary examinations performed to maximize anatomical detail while maintaining clinically acceptable acquisition times in both MRI systems. In the high-field system T2-weighted fast spin-echo (FSE 2D) and T1-weighted fast spin-echo fat-saturated (FSE 2D FatSAT) sequences were used, while in the low-field scanner a gradient-echo sequence (balanced fast field echo 3D: B-FFE 3D) was selected for the study. Table 1 summarizes the main acquisition parameters used with high- and low-field MRI systems.

Although an attempt was made to acquire whole-body images using a long surface coil on the high-field MRI system (Figure 1), the resulting image resolution was not considered adequate.

Therefore, the cranial, middle, and caudal thirds of each animal were imaged separately on both MRI systems. For each anatomical region examined, images were acquired in the transverse, sagittal, and dorsal planes.

Sequence parameters (repetition time, echo time, field of view, slice thickness and matrix) were adjusted to the body size of each animal, aiming at an in-plane resolution between 0.15 and 0.6 mm and a slice thickness of 1.5–3.0 mm (Table 1). Total examination time per animal ranged from 60 to 75 min approximately.

### 2.4. Image Analysis

MRI datasets were transferred to a dedicated workstation in DICOM format and were analyzed using the Radiant DICOM Viewer (Medixant. Radiant DICOM Viewer, Version 2025.1, URL (accessed on 28 November 2025: https://www.radiantviewer.com). Three researchers with broad experience in reptile diagnostic imaging and morphology evaluated the images in consensus. MRI findings were systematically correlated with the recently described anatomy of this species, published by the same research group in a study that correlated anatomical dissection and CT scans of the same animals [30]. Thus, because the study was descriptive and anatomical, no formal interobserver agreement analysis was performed.

## 3. Results

Although the MRI exams were performed by dividing the anatomy of each animal into three sections, in order to facilitate the description of the anatomy, the structures will be described following a cranial-to-caudal anatomical order, focusing particularly on the soft tissues. Superficial structures such as the osteoderms and the lateral groove were easily identified in both high-field and low-field MRI images (Figure 2), although the grade of definition was better in the first, especially when T2-weighted FSE 2D sequence was used.

In the rostral aspect of the head, the nasal cavity and the ethmoturbinates were clearly visible in the dorsal and transverse planes. The turbinate appeared as medium signal intensity septae, with air in between. Otic clefs were also observed as very small defects in the osteoderms superficial covering. Eyes presented nearly spherical with hyperintense fluid content and centrally located hypointense lens. A hyperintense linear structure covering partially the eyes was considered consistent with the third eyelid. All these structures were correctly identified in both high-field and low-field MRI images, although the quality of the former was better due to the higher signal-to-noise ratio and better spatial resolution (Figure 3).

Nervous system structures identified in this study were the brain, the spinal cord and the optic nerves. Although one might expect brain images to show significantly superior anatomical detail in high-field images compared with low-field images, in this study they appeared quite similar. The olfactory bulb, cortex, optic tectum, cerebellum, fourth ventricle and rhombencephalon could be distinguished with both systems. The spinal cord, however, was identified quite clearly on the T2-weighted FSE 2D images obtained with the high-field scanner, but it could not be clearly distinguished on the low-field images. Conversely, both optic nerves were identified using the gradient-echo sequence on the low-field MRI but not on the high-field images, although this difference was likely due to the fact that the imaging plane was not aligned with the course of the optic nerves in the MRI examinations performed on the high-field scanner (Figure 4).

Other structures identified in the head included the tongue, the pterygoid muscles, the mandible, teeth, part of the hyoid bone, and the opening of the vomeronasal organ and choanae. The tongue showed homogeneous intermediate signal intensity, slightly higher than that of the pterygoid muscles, which were located just caudal to the tongue. A slight narrowing of the passage between oral and pharyngeal cavities, limited by fine hyperintense convex margins, was considered consistent with the glottis, which could be identified in all three planes: transverse, sagittal, and dorsal. Cranial to the glottis, the opening of the vomeronasal organ and choanae was identified as a hypointense band in the dorsal plane and as a triangular structure in the transverse plane. The mandible showed a hyperintense central band (medullary cavity) surrounded by fine hypointense lines (cortical bone). The teeth appeared as very small hypointense structures aligned with both hemimandibles. Part of the hyoid bone could be recognized only in the transverse plane, as a paired structure with hypointense peripheral margin and hyperintense central area. All these structures were observed in both high-field and low-field images, although the quality of the former was better (Figure 5).

One of the most difficult structures to identify was the thyroid gland. This was observed only on the T2-weighted FSE 2D images of just one male specimen. The images were obtained with the high-field system and the gland was visible only in the transverse plane. It appeared as an oval structure of 2.1 mm of maximum diameter, slightly hyperintense compared with near muscles. It was located just caudal to the glottis (Figure 6).

In both high-field and low-field images, the heart was clearly identified. The ventricular wall showed intermediate signal intensity, slightly hyperintense compared with the axial muscles. The blood within the ventricular lumen appeared hypointense on the T2-weighted FSE 2D high-field images and hyperintense on the gradient-echo B-FFE 3D low-field images. The atria were slightly hyperintense on the T2-weighted FSE 2D high-field images and markedly hyperintense on the gradient-echo B-FFE 3D low-field images when compared with the ventricular wall. The sinus venosus was located to the left of the atria and showed similar signal intensity, although with a smaller size (Figure 7).

It was possible to visualize the initial course of the two cranial cava veins, which joined the sinus venosus, on both the T2-weighted FSE 2D and T1-weighted FSE FatSAT high-field images. In the former, they appeared as hypointense tubular structures in the sagittal plane and as rounded structures in the transverse plane, whereas in the latter, they were identified only in the transverse plane as hyperintense rounded structures. The aortic arches were also visualized on both the T2-weighted FSE 2D and T1-weighted FSE FatSAT high-field images. On the T2-weighted images, they appeared as hypointense tubular structures located just cranial to the atria and were visible only in the dorsal plane, whereas on the T1-weighted images, they showed very high signal intensity and were observed only in the transverse plane (Figure 8).

Other major vessels identified were the dorsal aorta, the caudal vena cava and the dorsal vein of the tail. All of them were identified in the T2-weighted FSE 2D high-field images as hypointense structures, rounded-shaped in the transverse plane (Figure 9) and tubular-shaped in the sagittal and dorsal planes (Figure 10).

The iliac vein bifurcation was also seen in the dorsal and transverse plane in T2-weighted FSE 2D high-field images (Figure 11).

In B-FFE 3D low-field images, the dorsal aorta was the only large vessel that could be identified. It was observed in transverse and dorsal planes, poorly defined (Figure 12).

In relation to the respiratory system, the trachea, tracheal bifurcation, and lungs were easily identified due to their air content, which produces no signal and therefore appears black in the MRI images. The trachea appeared as a tubular structure located rostrally along the midline, although it curves slightly to the right as it passes near the heart before returning to the midline at the tracheal bifurcation. The right lung was longer than the left lung in all animals. Two animals showed fine septa within the left lung. The trachea was best visualized in the transverse and sagittal planes. In the dorsal plane, it was not possible to visualize the entire course of the trachea in a single image due to its slightly undulating course. The sagittal plane did not allow visualization of both lungs in the same image for direct comparison, in contrast to the transverse and dorsal planes, which were considered more adequate for their evaluation. Both the high-field (Figure 13) and low-field (Figure 14) images provided good definition of these structures.

A distinctive anatomical structure identified in all these animals was a large air-filled chamber located just dorsal to the cranial half of the trachea. It was not possible to identify a direct connection between this chamber and either the trachea or the lungs on the images, so the term “cervical air chamber” will be used (Figure 15).

Regarding the digestive system, the esophagus was identified as a long tubular structure, best visualized in the transverse and midsagittal planes, with two layers of intermediate signal intensity, although the inner layer was slightly hyperintense compared with the outer layer. Some intraluminal content was also observed, consisting mainly of gas and mixed-signal intensity ingesta. The esophagus courses between the lungs, ventral to the dorsal aorta and dorsal to the trachea, tracheal bifurcation, and liver. These structures served as anatomical landmarks for their identification, particularly in the low-field images, due to the lower anatomical detail (Figure 16).

The stomach showed a similar appearance to the esophagus, although with a slightly larger transverse diameter. The three imaging planes (transverse, sagittal, and dorsal) provided good visualization of its location. Cardia and pylorus were identified as areas of luminal narrowing, connecting cranially with the esophagus and caudally with the small intestine. These narrowings were best appreciated in the sagittal and dorsal planes. On both T2-weighted FSE 2D high-field images and gradient-echo B-FFE 3D images, a slightly hyperintense inner layer and a hypointense outer layer could be distinguished, as in the esophagus. In some animals, the inner layer exhibited mucosal folds, which became less evident as gastric distension increased. The gastric intraluminal content showed variable signal characteristics, ranging from gas and markedly hypointense structures (possibly of mineral density) to hyperintense material, such as fluid (Figure 17).

The small intestine was more difficult to distinguish and appeared as multiple rounded-to-oval structures located between the stomach and the large intestine. This appearance was similar in the transverse, sagittal, and dorsal planes due to its tortuous course and the concentration of the intestinal loops within a relatively small portion of the coelomic cavity. In this intestinal segment, differentiation between the intestinal wall and the intraluminal content was more challenging in both high-field and low-field images, particularly in the latter because of the lower spatial resolution and signal-to-noise ratio. The large intestine was readily recognized because of its longer course through the caudal part of the coelomic cavity and its greater transverse diameter compared with the other segments of the digestive tract. On T2-weighted FSE 2D high-field images, it was well visualized in all three planes (transverse, sagittal, and dorsal), with a thin, well-defined hypointense wall. In contrast, on gradient-echo B-FFE 3D images, the sagittal and dorsal planes were more useful for complete evaluation, as the lower anatomical detail relative to the high-field images was more apparent in the transverse plane. The intraluminal content included larger amounts of gas (signal void) than the esophagus and stomach, together with variable amounts of hyperintense and hypointense material (Figure 18).

The liver appeared as a relatively elongated organ located approximately within the middle third of the coelomic cavity, occupying the right side and central portion of the ventral half of the cavity, immediately ventral to the right lung. Its caudal margin reached approximately the mid-portion of the stomach, which lay to the left of the liver. T2-weighted FSE 2D high-field images provided superior anatomical detail of the organ. In this sequence, the hepatic parenchyma appeared markedly hypointense, and two hyperintense vessels were observed coursing longitudinally through the liver. By contrast, on gradient-echo B-FFE 3D low-field images, the liver appeared moderately hyperintense, and no intrahepatic vessels could be identified. In both high-field and low-field images, the liver was more easily identified and evaluated in its entirety on the transverse and sagittal planes, whereas the dorsal plane should be perfectly alienated with the organ to provide useful information. The gallbladder was clearly identified on T2-weighted FSE 2D high-field images as an oval hyperintense structure, with a craniocaudal length ranging from 6.0 to 7.3 mm and a width of 3.0 to 4.3 mm. It was also visible on gradient-echo B-FFE 3D low-field images, although it exhibited lower contrast because of the high signal intensity of the surrounding hepatic parenchyma. As observed for the hepatic parenchyma, the transverse and sagittal planes were the most useful for gallbladder identification and evaluation (Figure 19).

The pancreas was identified only on T2-weighted FSE 2D high-field images and could not be visualized on gradient-echo B-FFE 3D low-field images. On T2-weighted FSE 2D high-field images, it appeared as an elongated hypointense organ, slightly hyperintense relative to the hepatic parenchyma. It was located between the caudal aspect of the liver and the stomach, extending along the medial margin of the stomach and ending immediately ventral to it. The transverse plane allowed the course of the pancreas to be followed slice by slice (Figure 20). It was not identified on sagittal images and was visible on dorsal images in only one animal, probably because of its small transverse diameter.

Like the pancreas, the spleen was identified only on T2-weighted FSE 2D high-field images and could not be visualized on gradient-echo B-FFE 3D low-field images. It appeared as a small, oval-shaped hypointense organ that was visualized only on the transverse plane, where it was visualized between the pancreas and the stomach (Figure 20B). It could not be identified on either the sagittal or dorsal planes.

Kidneys appeared as very elongated, quite flat bilateral structures located in the most dorsal part of the caudal third of the coelomic cavity, on either side of the dorsal aorta. Although they were visible in both T2-weighted FSE 2D high-field images and gradient-echo B-FFE 3D low-field images, they were much easier to identify with the high-field system. In the T2-weighted FSE 2D high-field images, the kidneys exhibited intermediate signal intensity and were slightly hyperintense relative to the adjacent muscles. Among the images acquired with the high-field system, the transverse plane proved to be the most useful. In the sagittal plane, only the right kidney of one specimen could be adequately identified, whereas neither kidney could be adequately visualized in the dorsal plane. In the gradient-echo B-FFE 3D low-field images, only the right kidney of one specimen could be identified, and only in the sagittal and dorsal planes, but not in the transverse plane. In these images, the kidney exhibited relatively high signal intensity, similar to that of the adjacent muscles. The kidneys proved to be the best anatomical landmark for locating the testes, which were situated immediately ventral to them. The testes exhibited tubular morphology and followed a course parallel to that of the kidneys, with a transverse diameter ranging from 2 to 3 mm depending on the specimen. They were identified only in the high-field images, in which they exhibited markedly lower signal intensity than the kidneys in the T2-weighted FSE 2D images. Only a portion of one testis could be identified in the sagittal plane of a single specimen, whereas the testes could not be visualized in the dorsal plane in any of the animals (Figure 21).

Ovarian follicles were very difficult to identify in the gradient-echo B-FFE 3D low-field images. They appeared as small, rounded structures with a hypointense peripheral band and a more hyperintense central area (Figure 22). In this study, the female specimen could not be examined with the high-field system; therefore, their imaging characteristics under this modality could not be described.

Hemipenes were identified only in the T2-weighted FSE 2D high-field images. They appeared as paired, elongated tubular structures located immediately caudal to the cloaca and ventral to the vertebral column, in the proximal portion of the tail. They exhibited low signal intensity, similar to that of the adjacent muscles. The transverse plane was the most useful for their evaluation, whereas visualization was more difficult in the sagittal and dorsal planes (Figure 23).

The cloaca was easily identified in both the high-field and low-field images at the caudal extremity of the coelomic cavity. The three imaging planes (transverse, sagittal, and dorsal) were useful for its visualization. In both the T2-weighted FSE 2D high-field images and the gradient-echo B-FFE 3D low-field images, part of the intraluminal content appeared hyperintense, consistent with fluid, whereas very low-signal oval structures of variable size were also observed within the lumen, compatible with urate salts. In both image sets, a thin hypointense cloacal wall could be distinguished in all three planes, although it was depicted with greater detail in the high-field images (Figure 24).

Table 2 summarizes the anatomical structures identified with each of the MRI systems used for this study.

## 4. Discussion

Despite the increasing use of MRI in reptile medicine, whole-body MRI anatomical descriptions remain scarce, particularly in elongated squamates. In reptile medicine and surgery, advanced imaging techniques have gained relevance over the past two decades, both in veterinary clinical practice and zoology [32]. A study published by Kuoni et al. in 1993 is considered one of the first scientific journal publications specifically dedicated to the use of magnetic resonance imaging in reptiles, in which the authors presented practical experiences with tortoises [33]. The *Pseudopus apodus* is an uncommon species, so the available anatomical information is quite limited. Recently, the dissectional and computed tomography (CT) anatomy of this species has been described [30] by part of the authors of the present study. Here, its MRI anatomy using both high-field and low-field systems is presented.

Although low-field MRI scanners are generally considered less suitable for obtaining detailed images of small anatomical structures, such as those of *Pseudopus apodus*, the low-field system used in this study, together with the selected gradient-echo B-FFE 3D sequence, provided good anatomical detail for most soft tissue organs of the head, coelomic cavity, and tail. However, the spinal cord, thyroid gland, most blood vessels, spleen, pancreas, testes, and hemipenes were visualized only with the high-field MRI system. The only small soft tissue structures identified in the low-field images but not in high-field images were the optic nerves and the ovarian follicles.

Regarding the nervous system, the low-field images provided good visualization of the brain, as in the high-field images, although the latter provided superior image quality. The most likely explanation for the visualization of the optic nerves only with the low-field system is that the greater slice thickness used in this MRI protocol, together with the exact alignment of the imaging plane with the course of these nerves, enabled their complete visualization, whereas this was not achieved with the high-field system. This finding therefore should not be interpreted as an intrinsic superiority of the low-field system for optic nerve evaluation.

Numerous studies have used high-field MRI to describe reptilian brain anatomy. In 2000, Anderson et al. used high-field MRI to describe the brain of the garter snake, with particular emphasis on vomeronasal and olfactory systems [12]. In 2012, Arencibia et al. described the sectional and MRI anatomy of the head, including the brain, of juvenile loggerhead sea turtles (*Caretta caretta*) using five female cadavers scanned with a 1.5 T MRI system [14]. In 2018, Hoops et al. published a three-dimensional MRI-based atlas of the Australian tawny dragon brain using a non-clinical ultra-high-field 11.74 T MRI system [16]. Later, in 2021, the same type of magnet was used to publish a fully segmented three-dimensional anatomical atlas based on the scanning of 13 heads of male Australian tawny dragons [18]. In 2020, Billings et al. published a three-dimensional digital atlas of the forebrain of the Nile crocodile using a single cadaver scanned with a 7 T MRI system [17]. In 2022, Foss et al. developed an MRI-based imaging protocol and brain atlas for the bearded dragon (*Pogona vitticeps*) using a 3 T MRI system [19]. In 2023, González Rodríguez et al. described the MRI anatomy of the head of the rhinoceros iguana (*Cyclura cornuta cornuta*) using two adult female carcasses scanned with a 1.5 T MRI system [21]. More recently, in 2024, Jiménez et al. used a 4.7 T MRI system to compare the forebrain anatomy of the turtle *Trachemys scripta* (order Testudines), the lizard *Pogona vitticeps* (order Squamata), and the snake *Python regius* (order Squamata) [22]. In 2025, the same field strength (4.7 T) was used to provide an in-depth neuroanatomical characterization of the brain of *Pseudopus apodus* by combining MRI with immunofluorescence-based histological analysis of four carcasses [23]. To our knowledge, the present study is the first to describe the brain anatomy of *Pseudopus apodus* using clinical low-field (0.23 T) and high-field (1.5 T) MRI. Although the image quality is lower than that obtained with 3 T or higher-field MRI systems, the principal neuroanatomical structures could still be identified. This expands the currently available MRI anatomical information for elongated squamates using MRI systems that are accessible in clinical practice.

In contrast, the spinal cord could not be identified in the MRI low-field images of the *Pseudopus apodus* in this work, probably because the spatial resolution was not enough for its correct visualization. In the T2-weighted FSE 2D high-field images, the entire course of the spinal cord within the vertebral canal was visualized in the transverse plane. The sagittal and dorsal planes were less useful, because achieving optimal alignment of the entire spinal cord with the imaging plane was very difficult. Although MRI has been proposed as a valuable imaging modality for assessing spinal cord injury in reptiles [32], the size of the animal remains the main limitation to make this evaluation possible, and this limitation is particularly relevant in *Pseudopus apodus* because of its small spinal cord diameter. As example, four cases of cervical compressive myelopathy in Komodo dragons (*Varanus komodoensis*) were reported by Dawn et al. [34], but the mean body weight of this species is approximately 70 kg. In a recent study in which high-field MRI was performed in three chelonians species, the authors indicate that the ability to visualize the spinal cord and brain was linked to the size of the animals, so in the smaller turtles (<15 cm), only the cerebrospinal signal intensity could be identified but not the spinal cord [25].

Like the spinal cord, most blood vessels could not be identified with the low-field MRI system. In a recent study, several large caliber vessels, such as the right and left aortic arches, the abdominal aorta, the caudal vena cava, the portal vein, the right and left hepatic veins, and the gastric artery were identified with a 1.5 T MRI system in three species of quelonians (*Trachemys scripta*, *Testudo marginata* and *Testudo hermanni*) [25]. Additional vessels such as precaval and postcaval veins, right and left pulmonary veins, the pulmonary trunk and right and left pulmonary arteries were also identified in European pond turtles with a 3 T MRI system [26]. In the previous CT study of *Pseudopus apodus* published by part of the authors of the present work, that will be named as “the previous clinical CT study of the *Pseudopus apodus*” in the rest of this discussion for comparative purposes, only the aortic arches, dorsal aorta, and cranial cava veins could be identified, and the visualization of these vascular structures improved after intravenous iodinated contrast administration [30].

The heart was clearly identified in both high-field and low-field MRI images. In the previous clinical CT study of the *Pseudopus apodus* it was identified in pre-contrast images, although intravenous iodinated contrast administration improved the visualization of the heart [30]. In 2009, Mitchell proposed CT and MRI as useful imaging modalities for assessing cardiac disease in reptiles, although he also highlighted the high cost of these procedures and the limited availability of these imaging modalities to general practitioners as important limitations of these diagnostic tools [35]. More recently, cardiac ejection fraction has been evaluated in red-footed tortoises (*Chelonoidis carbonarius*) using a 1.5-T MRI system [36], and the MRI anatomy of the heart in the leatherback turtle has been described using a 3 T MRI system [20].

For the high-field MRI protocol, a T2-weighted FSE 2D and a T1-weighted FSE FatSAT sequence were selected. However, the latter provided a lower signal-to-noise ratio, lower spatial resolution, and poorer soft tissue contrast than the former. Despite these limitations, it was useful as complementary sequence for vascular evaluation, as blood vessels exhibited very high signal intensity in the T1-weighted FSE FatSat images, resulting in excellent contrast with the surrounding soft tissues. For all other anatomical structures described in this study, the T2-weighted FSE 2D sequence provided clearly superior image quality. Consequently, the anatomical descriptions presented here are based on the images acquired with this sequence.

Both the high-field and low-field images provided good visualization of the respiratory system, including the trachea, tracheal bifurcation, and lungs. There were no marked differences in anatomical detail comparing both sets of images, which could be attributable to their high air content and limited soft tissue component. In line with previous reports on pulmonary asymmetry in serpentiform lizards [6,37], MRI confirmed a reduction in length of the left lung compared to the right in *Pseudopus apodus*. These findings were quite similar to those reported for clinical CT in this species [30].

MRI has been used to identify pathological changes in the respiratory tract in chelonians [38]. Pees et al. (2006) reported a comparative study using CT and MRI for evaluating pneumonia in three Indian phytons (*Phyton molurus*) and demonstrated the high diagnostic value of both imaging techniques for the diagnosis of respiratory diseases in snakes [39]. The trachea and lung fields, including pulmonary septa, can readily be identified by MRI, and pulmonary changes such as edema, inflammatory conditions, and interstitial changes can also be detected with this imaging modality [40]. However, the long time of acquisition compared with other techniques, like radiography or CT, limits the use of MRI in patients with dyspnea or poor clinical condition [41].

No direct connection between the air-filled cervical structure, referred to as “cervical air chamber” in this work, and the trachea or lungs could be demonstrated by MRI. Nevertheless, its location and air-filled nature raise the possibility that this structure may be anatomically or functionally comparable to the cervical air sacs or related air-containing diverticula described in birds. Air-filled pulmonary diverticula reminiscent of avian air sacs have also been described in several squamate reptiles [42,43]. However, to our knowledge, no equivalent structure has been specifically characterized in *Pseudopus apodus*. Therefore, any interpretation in terms of homology with the avian respiratory system remains speculative, and further anatomical and functional studies would be required to clarify the origin and significance of this structure.

Esophagus, stomach, small intestine and large intestine were all clearly identified in both high-field and low-field images, although the anatomical detail was superior in high-field images. Nevertheless, only the T2-weighted FSE 2D high-field images allowed differentiation of a slightly hyperintense inner layer and a hypointense outer layer in the walls of the esophagus and stomach. In the stomach, the inner layer exhibited mucosal folds, which became less evident as gastric distension increased. Gastric rugae in reptiles may be prominent or absent [44]. Arencibia et al. described the stomach of loggerhead sea turtles (*Caretta caretta*) in transverse T2-weighted MRI as a rounded hyperintense structure surrounded by a thin hypointense muscular wall; this description can be interpreted as no layering distinction of the gastric wall, since the hyperintensity probably corresponds to the fluid intraluminal content [24]. Di Ianni et al., in their high-field MRI study of the coelomic organs in three species of chelonians, reported that it was not possible to distinguish subdivision into layers in the wall of the stomach and described the wall of the esophagus as slightly hyperintense compared to the limb muscle using several MRI sequences [25]. Zehtabvar et al., using a 3 T MRI system, also described the gastric wall of the European pond turtle as appearing hyperintense compared with the liver [26]. In contrast, a previous MRI study performed with turtles indicated that, in general, the layers of the wall of the digestive tract were seen and the submucosal layer of the esophagus was more visible because of the hyperintensity of the papillae in the T2-weighted image [13], although a more detailed description would be needed to establish a more exhaustive comparison with the results of our study.

The distinction between the small intestine wall and its intraluminal content was more challenging with both high-field and low-field MRI systems in this work, particularly in low-field images. The wall of the large intestine appeared as a thin hypointense layer, which was more clearly defined in the high-field images. Di Ianni et al. indicated that the intestinal wall was not always clearly visible in the three species of chelonians that they studied with high-field MRI [25]. In the previous clinical CT study of *Pseudopus apodus*, these organs were more readily identified when they contained intraluminal gas or hyperattenuating content. However, differentiating segments of the digestive tract with attenuation similar to that of the surrounding coelomic soft tissues was more challenging. Furthermore, CT images did not allow the identification of distinct layers within the esophageal and gastric walls [30].

The high-field images provided superior anatomical detail of the liver compared with low-field images, although with both MRI systems the gallbladder was easily identified. In contrast, intrahepatic vessels could only be identified in the high-field images. In another reptile species, the green iguana, MRI allowed a presumptive diagnosis of hepatocellular carcinoma, although a liver biopsy was required to confirm the diagnosis [45]. In the previous clinical CT study of *Pseudopus apodus*, the liver could be identified, although the margins of the organ could not be clearly delineated in pre-contrast images. The gallbladder appeared in the CT as a small hypoattenuating structure surrounded by hepatic parenchyma. The difference in attenuation between them was more evident in post-contrast images [30].

The liver and the stomach served as anatomical landmarks for identifying the pancreas, due to its location between the caudal aspect of the liver and the stomach, extending along the medial margin of the latter and terminating immediately ventral to it. The pancreas was only visualized in the T2-weighted FSE 2D high-field images. Previous MRI studies in turtles indicated that the pancreas is a challenging organ to identify on MRI [13]. In an MRI study of European pond turtles (*Emys orbicularis*) with a mean body weight of 550 ± 10.22 g, the pancreas could not be identified despite the use of a high-resolution 3 T MRI system [26]. This organ was not identified in the previous CT study of *Pseudopus apodus* [30].

Increasingly, advanced imaging techniques such as CT and MRI are used in reptile gastroenterology, especially in patients where radiography and ultrasound are limited, as in many chelonian or crocodilian patients [46]. That would also be the case of the *Pseudopus apodus*, since the hardness of superficial osteoderms avoids the transmission of ultrasound waves [30], and the radiographs provide limited information (based on the author’s observations). MRI is usually considered only for select cases, because of the high price, anesthetic time, and low availability of equipment often limit its use in general practice [46].

The spleen was located between the pancreas and the stomach and was visualized only in the T2-weighted FSE 2D high-field images and in the transverse plane. Its small size made its identification challenging. The MRI anatomy of this organ in turtles has been described also as an oval to elongated structure, slightly hypointense compared to the liver [13]. This organ was not identified in the previous clinical CT study of *Pseudopus apodus* [30].

The kidneys were visible in both high-field and low-field images, although they were much easier to identify with the high-field MRI system. The transverse plane was considered the most adequate for their complete visualization. They were inconsistently visualized in the sagittal and dorsal planes. The ureters and urinary bladder were not seen in this work. Arencibia et al. identified the ureters and urinary bladder in loggerhead sea turtles (*Caretta caretta*) due to the high signal intensity of urine in the renal collecting system [24], although the size of this species is not comparable to that of the *Pseudopus apodus*. In previous studies conducted in turtles, ureters were not identified, and the urinary bladder was recognized only by the fluid content [13,15,25,26]. In the previous clinical CT study of *Pseudopus apodus*, kidneys were best seen in post-contrast images in transverse and dorsal planes, using the spine as an anatomical landmark, and neither the ureters nor the urinary bladder were visualized [30].

The testes were identified only in the high-field images, located just ventral to and running parallel to the kidneys. In contrast, they were not identified in the low-field images, probably because of the lower spatial resolution and poorer soft tissue contrast of this system compared with the high-field system. As with the kidneys, the transverse plane was considered the most suitable for its complete visualization. These structures were not identified in the previous clinical CT study of *Pseudopus apodus* [30].

The only female specimen included in the study was scanned exclusively with the low-field MRI system. Identifying the ovarian follicles with this system was challenging. A high-field MRI scanner would likely have provided more detailed images of the female gonads; however, this hypothesis could not be evaluated in the present study. It has been reported that ovarian follicles can be seen with MRI in chelonians [47]; depending on the stage of maturity, most of them were visualized as homogeneous round structures (hypointense to signal-free in T2-weighted images, and with higher intensity in T1-weighted images) or divided into layers of different intensities that were best seen in T2-weighted images [15,38,48], which is in agreement with the findings of the present work. MRI was successfully used to monitor follicular development in female veiled chameleons (*Chamaeleo calyptratus*). In that study, the smallest follicular structures detected by MRI measured 2 mm in diameter, as gastrointestinal contents and respiratory motion artifacts interfered with the identification of smaller follicles in some images [49]. It has been reported that, depending on the reptile species and developmental status, horizontal and concentric layering of various intensities can be observed in follicles and eggs using MRI. The pre-ovulatory follicles of lizards appear hyperintense in T2-weighted sequences and, in case of eggs, the albumen appears hyperintense, the yolk hypointense, and the calcified shell very hypointense in T2-weighted sequences, whereas in T1-weighted images the yolk appears of similar intensity as muscle [9,49,50]. Di Ianni et al. identified follicles only bigger than 5 mm in three species of chelonians with high-field MRI [25]. The ovarian follicles were also identified in the previous clinical CT study of *Pseudopus apodus*, but only in one of the two females scanned; they appeared as hypoattenuating rounded-to-oval structures, bounded by fine slightly hyperattenuanting margins [30].

In the field of reptile reproduction, MRI has been used for the examination of gravid specimens, such as a leopard tortoise (*Geochelone pardalis pardalis*) [8,47] and a Boa constrictor [51].

Hemipenes were identified in the proximal portion of the tail. Their similarity in signal intensity to that of the adjacent muscles made their identification challenging. Nevertheless, their paired tubular morphology and their position immediately caudal to the cloaca and ventral to the vertebral column served as landmarks to their correct identification, which was much easier in the transverse plane than in the sagittal and dorsal planes. These structures were not identified in the previous clinical CT study of *Pseudopus apodus* [30].

The cloaca was identified in both high-field and low-field images at the caudal extremity of the coelomic cavity. It had a thin hypointense wall, which was more clearly defined in the high-field images. In addition to fluid, very low-signal structures were also observed within the lumen, corresponding to urate salts, which is a common finding in these animals. In the previous clinical CT study of the *Pseudopus apodus*, the cloaca was also identified in the caudoventral coelom by the presence of hyperattenuating urate salts accumulated in its lumen, although the cloacal wall was not identified [30].

Bone structures of the head, especially mandible, nasal cavity and part of the hyoid bone, as well as vertebrae and ribs, were well-defined in both high-field and low-field MRI images, although the anatomical detail was superior in the former. Obviously, all these structures can be better evaluated in this species using clinical CT [30] or non-clinical micro-CT [2,3,4,5], but MRI can provide a first diagnostic approach for their clinical evaluation.

In 2013, Banzato et al. pointed out that the high cost of MRI equipment, the long acquisition times (particularly with low-field MRI), and the limited anatomical detail achievable in small patients were factors that could restrict the more widespread use of this technique in reptiles [11]. Our findings indicate that image quality has improved substantially since then, even with low-field MRI systems, allowing clinically useful information to be obtained from individuals with a small transverse body diameter, such as *Pseudopus apodus*.

This study has several important limitations. The main one was probably the low number of specimens, although it should be taken into consideration that this is an uncommon species with very limited availability for research. This study was designed to provide a descriptive MRI anatomical reference and not to characterize the full anatomical variability of the species, which would not be possible with this number of individuals. Further dissectional, cross-sectional, and MRI studies are needed to obtain more precise information, particularly regarding the soft tissue structures. Along the same lines, it is important to emphasize that, due to logistical constraints, the female specimen could only be scanned using the low-field system. Therefore, the descriptive information regarding the female gonads should be considered as a limited approximation. In addition, only one individual was examined with both MRI systems; therefore, differences observed between examinations should be interpreted descriptively and should not be considered as evidence of intrinsic differences in scanner performance.

Another limitation of this study was the lack of anatomical illustrations included in the manuscript. This is justified by the fact that the dissection studies of the animals evaluated in the present study had already been included in a previous publication [30] of the same research group, which is extensively cited throughout the present work. The reader may refer to the aforementioned publication for detailed information regarding these dissection studies.

The lack of contrast media administration can be considered a limitation of this work as well, particularly as a reference for future studies involving diseased animals. The use of gadolinium at doses of 1 to 2 mL/kg has been reported in several reptile species, with no detectable adverse effects reported [32].

Finally, it should be noted that the image quality obtained with both MRI systems was assessed qualitatively rather than using quantitative technical parameters. This approach was adopted because the primary objective of this work was to determine the anatomical structures that could be identified with each system and to characterize their location, morphology, signal intensity, and the imaging planes best suited for their assessment.

## 5. Conclusions

The present study shows that whole-body MRI is a feasible and informative tool for the anatomical evaluation of *Pseudopus apodus*. MRI provided excellent soft tissue contrast and was especially useful for assessing the central nervous system, visceral organs and gonads, although high density anatomical structures as bones, teeth and osteoderms were also adequately identified. These findings expand the available anatomical information for the species and provide an imaging-based reference for future clinical and comparative studies. Comparison with the previous CT-based study indicates that MRI offers superior soft-tissue contrast, especially for the brain, spinal cord, viscera, and gonads, whereas CT remains more suitable for the depiction of mineralized structures, osteoderms, and fine skeletal detail. Accordingly, both modalities should be regarded as complementary rather than interchangeable for anatomical and clinical applications in this species.

## Figures and Tables

**Figure 1:**
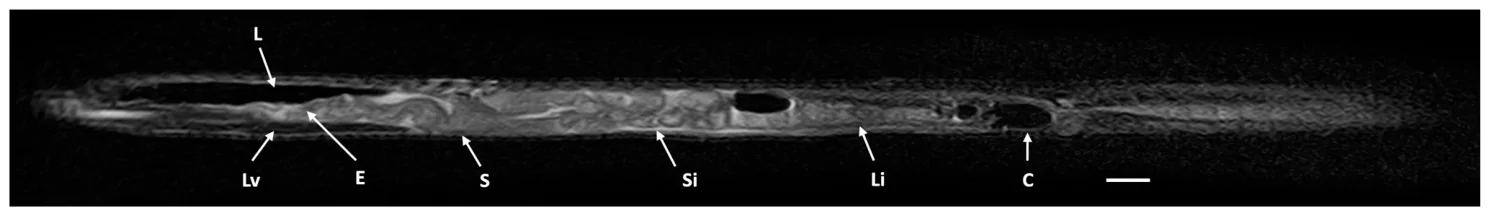
Representative whole-body MRI image of a *Pseudopus apodus* in sagittal plane obtained with the high-field system illustrating the limited spatial resolution obtained with a single acquisition. A T2-weighted FSE 2D sequence was used for the acquisition. The lung, liver, esophagus, stomach, small intestine, large intestine and cloaca are identified. See the list for abbreviations. Bar = 10 mm.

**Figure 2:**
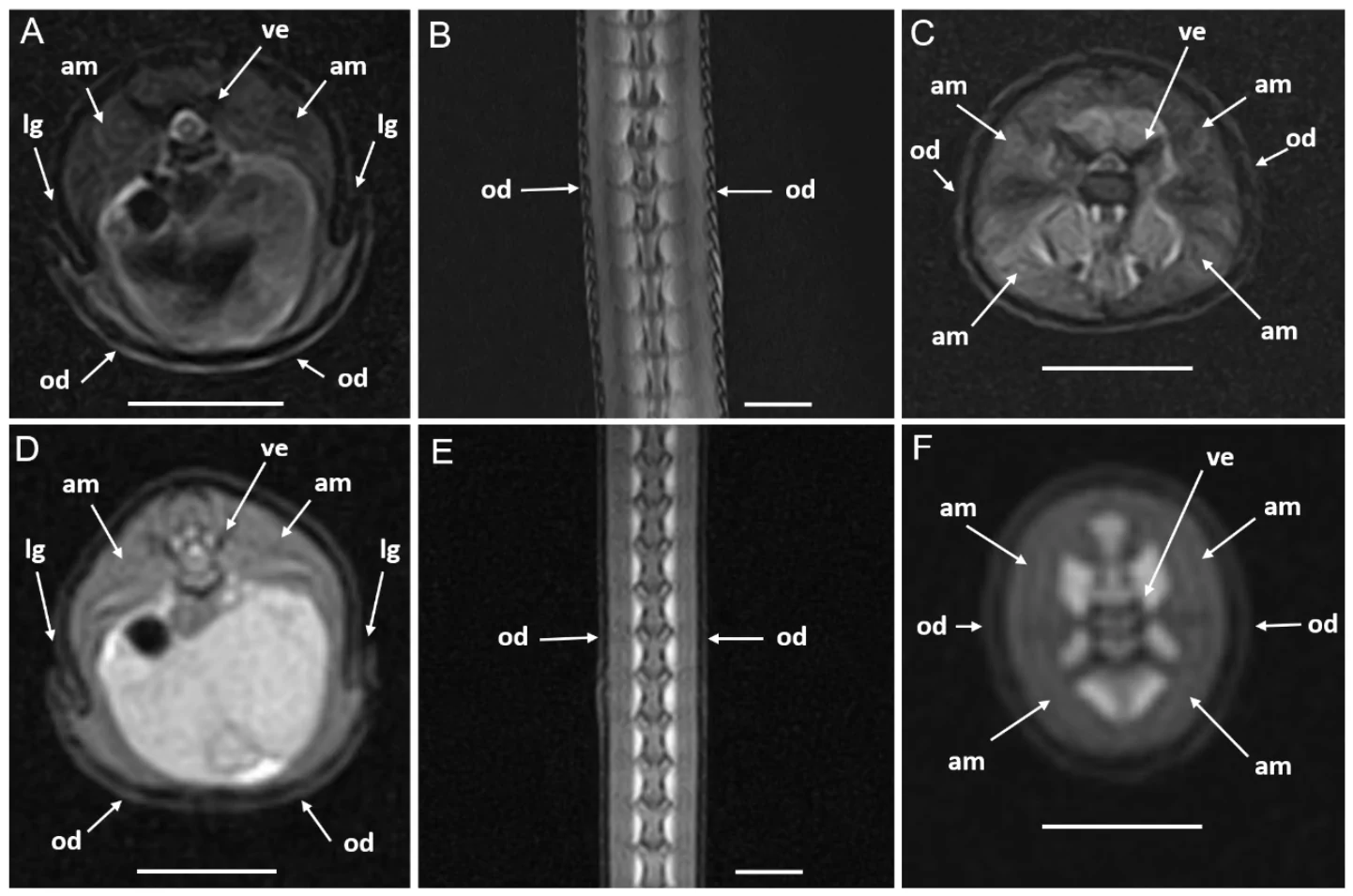
MRI images of a *Pseudopus apodus* in transverse (**A**,**C**,**D**,**F**) and dorsal (**B**,**E**) planes obtained with high-field (**A**–**C**) and low-field (**D**–**F**) systems. A T2-weighted FSE 2D sequence was used for images obtained with the high-field system and a gradient-echo B-FFE 3D sequence was employed for the low-field acquisition. Superficial osteoderms, axial muscles, vertebrae and lateral groove are identified. Images (**A**,**D**) were taken at the level of the heart, and (**B**,**C**,**E**,**F**) at the level of the cranial part of the tail. See the list for abbreviations. Bar = 10 mm.

**Figure 3:**
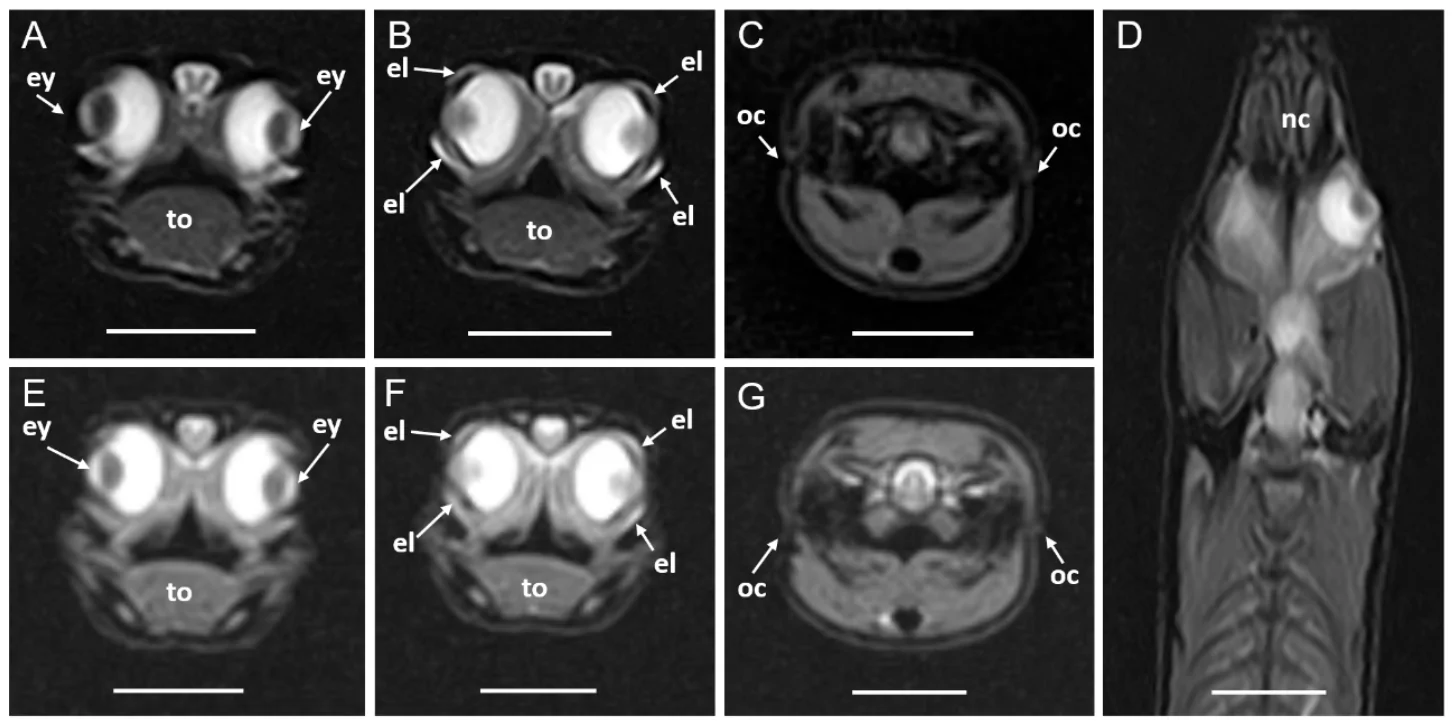
MRI images of a *Pseudopus apodus* in transverse (**A**–**C**,**E**–**G**) and dorsal (**D**) planes obtained with high-field (**A**–**D**) and low-field (**E**–**G**) systems. A T2-weighted FSE 2D sequence was used for images obtained with the high-field system and a gradient-echo B-FFE 3D sequence was employed for the low-field acquisition. Eyes, eyelids, tongue, otic clefts and nasal cavity are identified. See the list for abbreviations. Bar = 10 mm.

**Figure 4:**
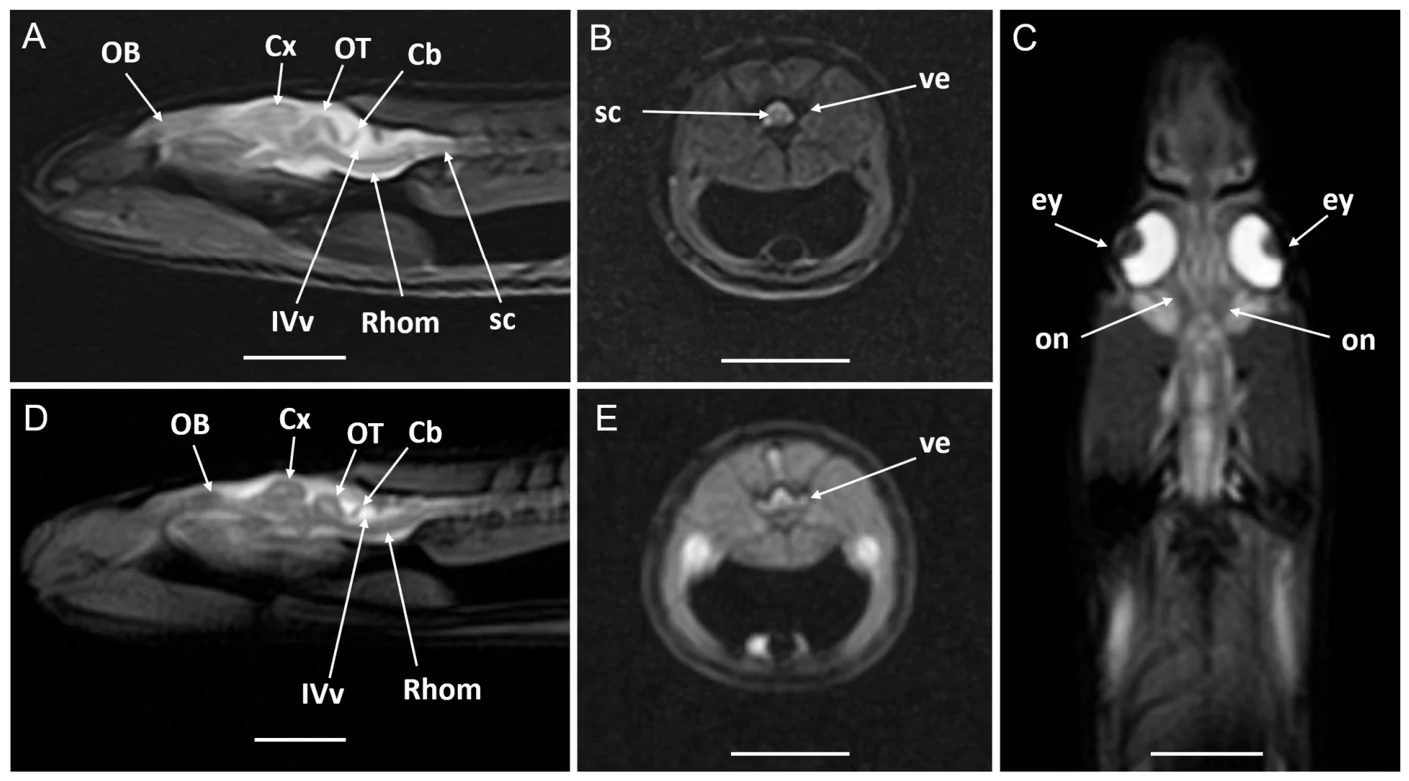
MRI images of a *Pseudopus apodus* in sagittal (**A**,**D**), transverse (**B**,**E**) and dorsal (**C**) planes obtained with high-field (**A**,**B**) and low-field (**C**–**E**) systems. A T2-weighted FSE 2D sequence was used for images obtained with the high-field system and a gradient-echo B-FFE 3D sequence was employed for the low-field acquisition. The olfactory bulb, cortex, optic tectum, cerebellum, fourth ventricle, rhombencephalon, spinal cord, vertebrae, eyes and optic nerves are identified. See the list for abbreviations. Bar = 10 mm.

**Figure 5:**
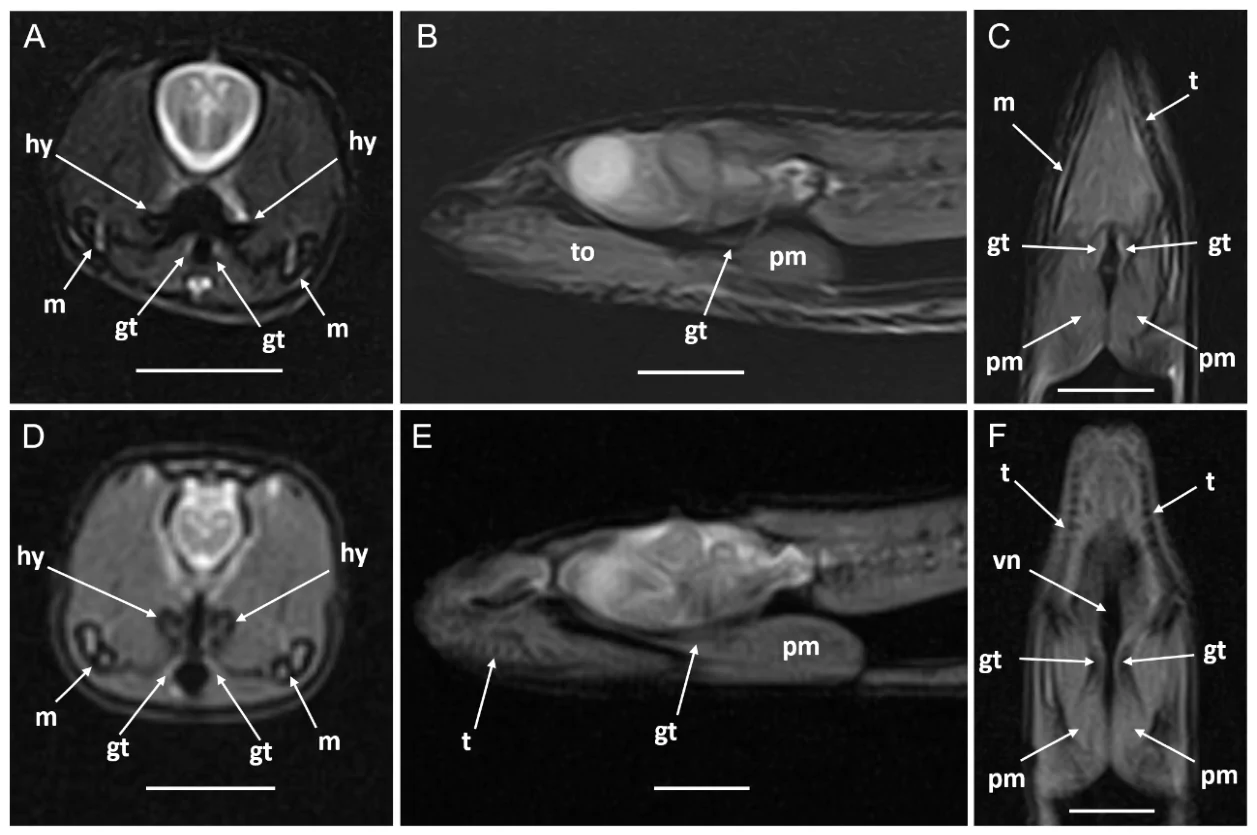
MRI images of a *Pseudopus apodus* in transverse (**A**,**D**), sagittal (**B**,**E**) and dorsal (**C**,**F**) planes obtained with high-field (**A**–**C**) and low-field (**D**–**F**) systems. A T2-weighted FSE 2D sequence was used for images obtained with the high-field system and a gradient-echo B-FFE 3D sequence was employed for the low-field acquisition. Glottis, hyoid bone, mandible, tongue, pterygoid muscles, teeth and opening of the vomeronasal organ and choanae are identified. See the list for abbreviations. Bar = 10 mm.

**Figure 6:**
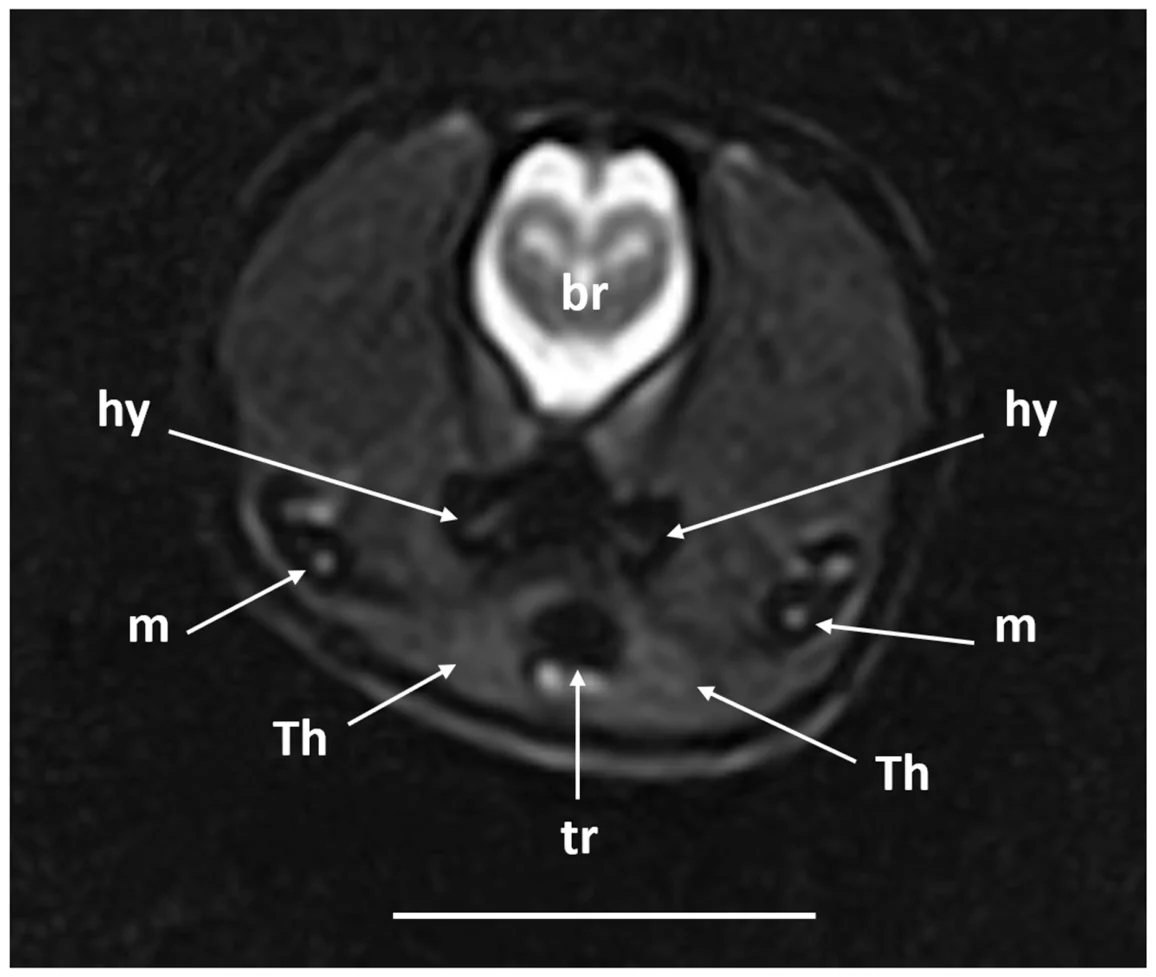
MRI of a *Pseudopus apodus* in transverse plane obtained with a high-field system. A T2-weighted FSE 2D sequence was used. Thyroid gland, trachea, hyoid bone, mandible and brain are identified. See the list for abbreviations. Bar = 10 mm.

**Figure 7:**
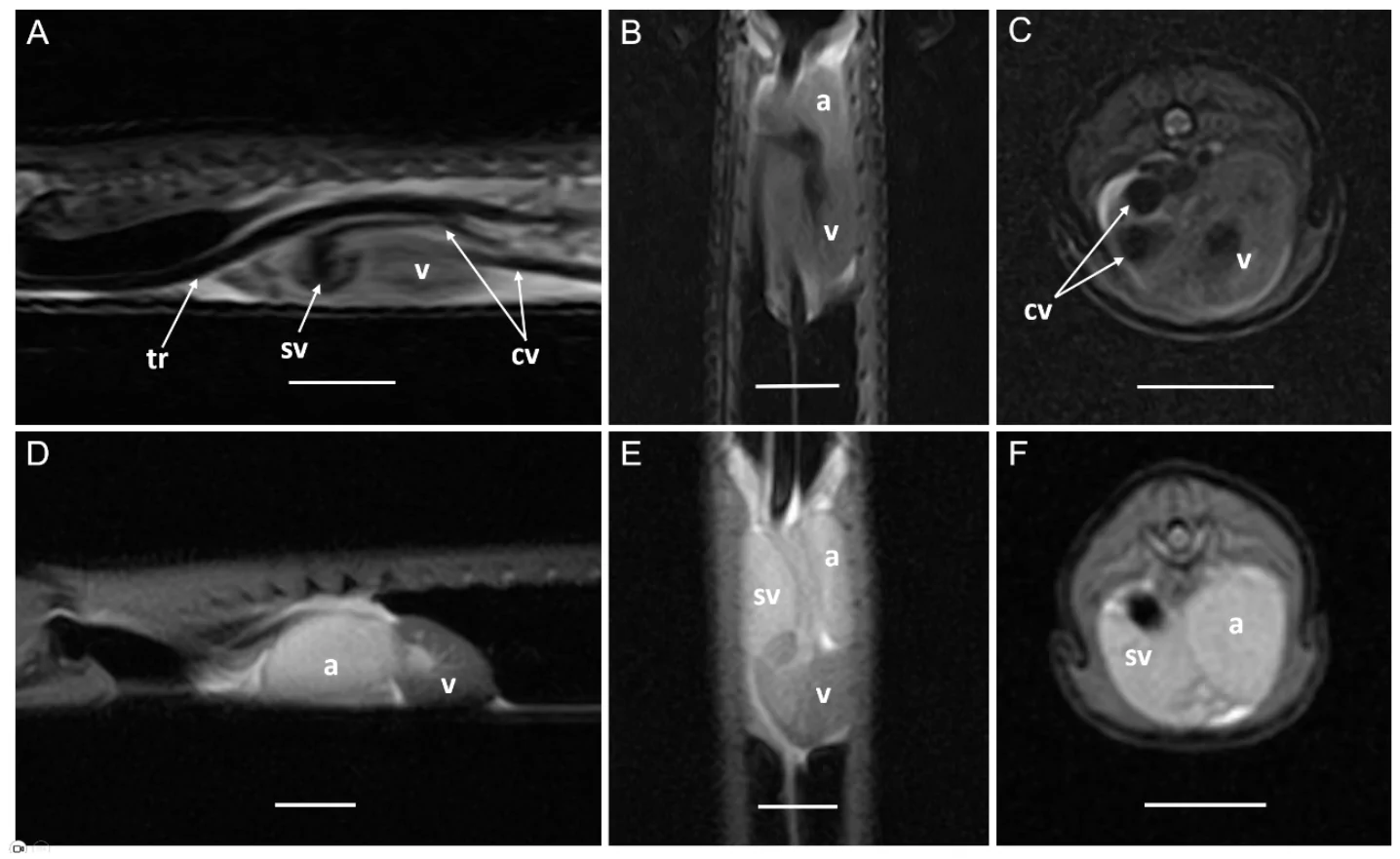
MRI images of a *Pseudopus apodus* in sagittal (**A**,**D**), dorsal (**B**,**E**) and transverse (**C**,**F**) planes obtained with high-field (**A**–**C**) and low-field (**D**–**F**) systems. A T2-weighted FSE 2D sequence was used for images obtained with the high-field system and a gradient-echo B-FFE 3D sequence was employed for the low-field acquisition. Atria, ventricle, sinus venosus, the two cranial cava veins and trachea are identified. See the list for abbreviations. Bar = 10 mm.

**Figure 8:**
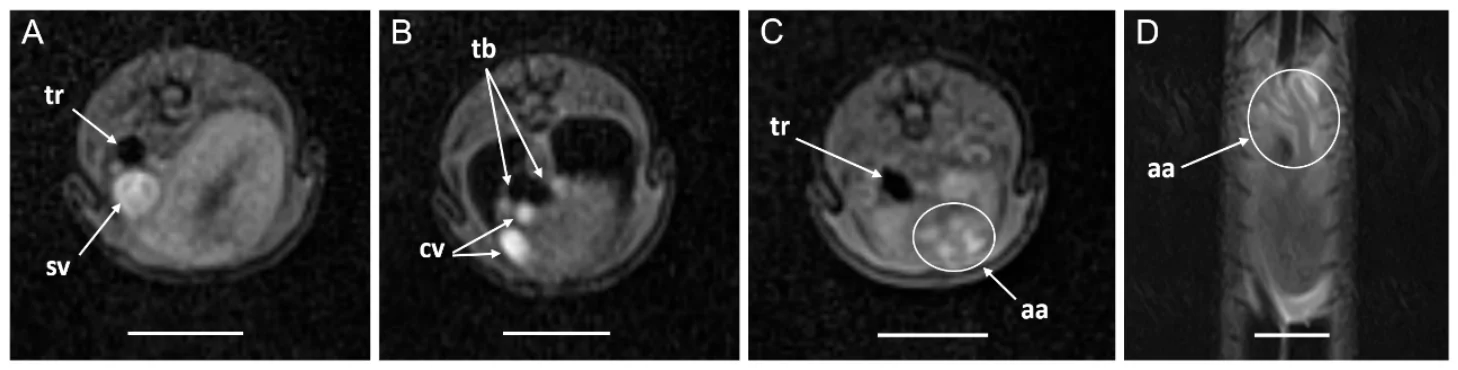
MRI images of a *Pseudopus apodus* in transverse (**A**–**C**) and dorsal (**D**) planes obtained with a high-field system. (**A**–**C**) were acquired with a T1-weighted FSE 2D FatSAT sequence and (**D**) with a T2-weighted FSE 2D sequence. Sinus venous, the two cranial cava veins, aortic arches, ventricle, trachea and tracheal bifurcation are identified. See the list for abbreviations. Bar = 10 mm.

**Figure 9:**
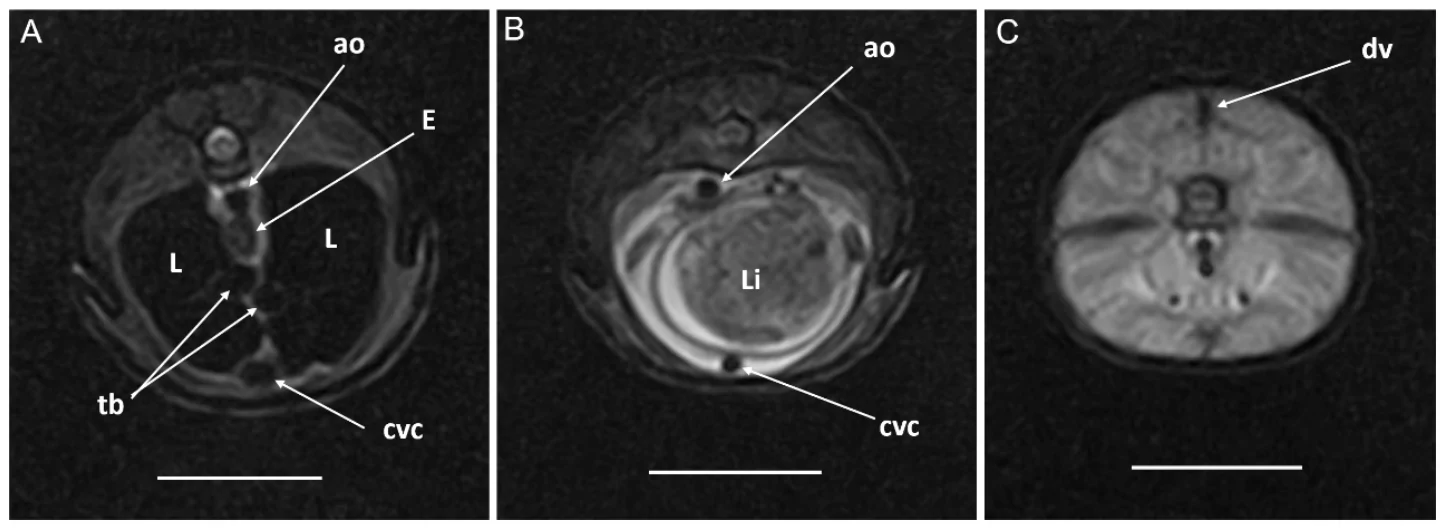
MRI images of a *Pseudopus apodus* in transverse plane obtained with a high-field system at the level of tracheal bifurcation (**A**), mid-part of large intestine (**B**) and cranial third of the tail (**C**). They were acquired with a T2-weighted FSE 2D sequence. Dorsal aorta, caudal vena cava, esophagus, tracheal bifurcation, lungs, large intestine and dorsal vein of the tail are identified. See the list for abbreviations. Bar = 10 mm.

**Figure 10:**
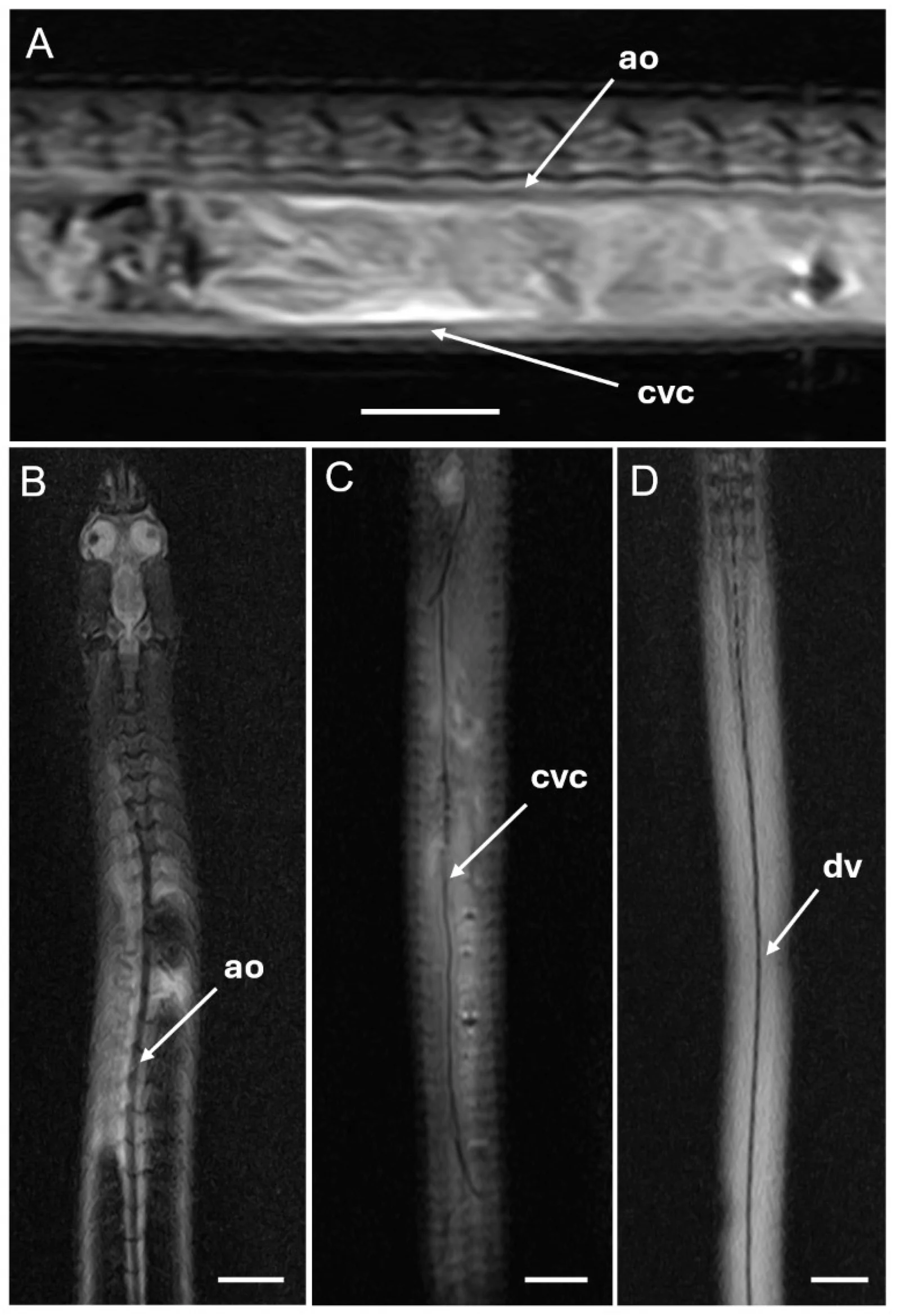
MRI images of a *Pseudopus apodus* in sagittal (**A**) and dorsal (**B**–**D**) planes obtained with a high-field system. They were acquired with a T2-weighted FSE 2D sequence. Dorsal aorta, caudal vena cava and dorsal vein of the tail are identified. See the list for abbreviations. Bar = 10 mm.

**Figure 11:**
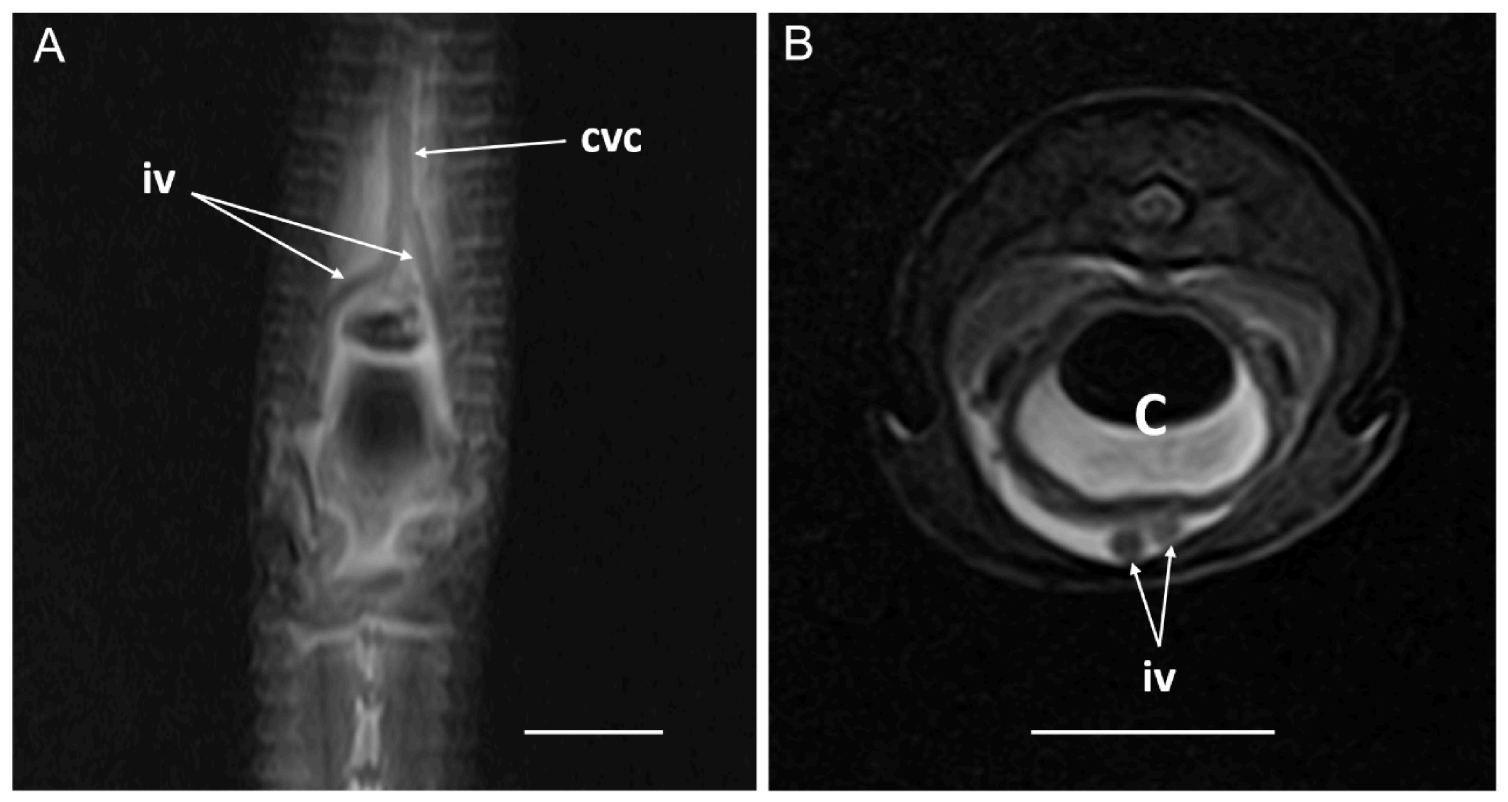
MRI images of a *Pseudopus apodus* in dorsal (**A**) and transverse (**B**) planes obtained with a high-field system. They were acquired with a T2-weighted FSE 2D sequence. Caudal vena cava, iliac veins bifurcation and cloaca are identified. See the list for abbreviations. Bar = 10 mm.

**Figure 12:**
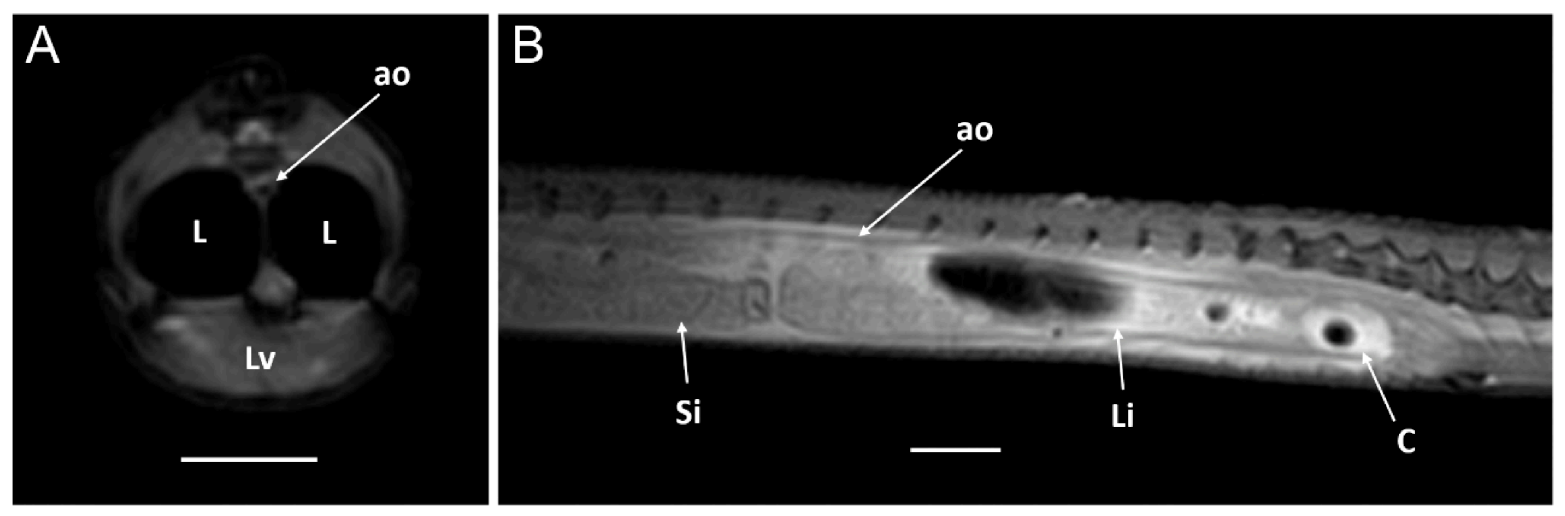
MRI images of a *Pseudopus apodus* in transverse (**A**) and sagittal (**B**) planes obtained with a low-field system. They were acquired with a gradient-echo B-FFE 3D sequence. Dorsal aorta, lungs, liver, small intestine, large intestine and cloaca are identified. See the list for abbreviations. Bar = 10 mm.

**Figure 13:**
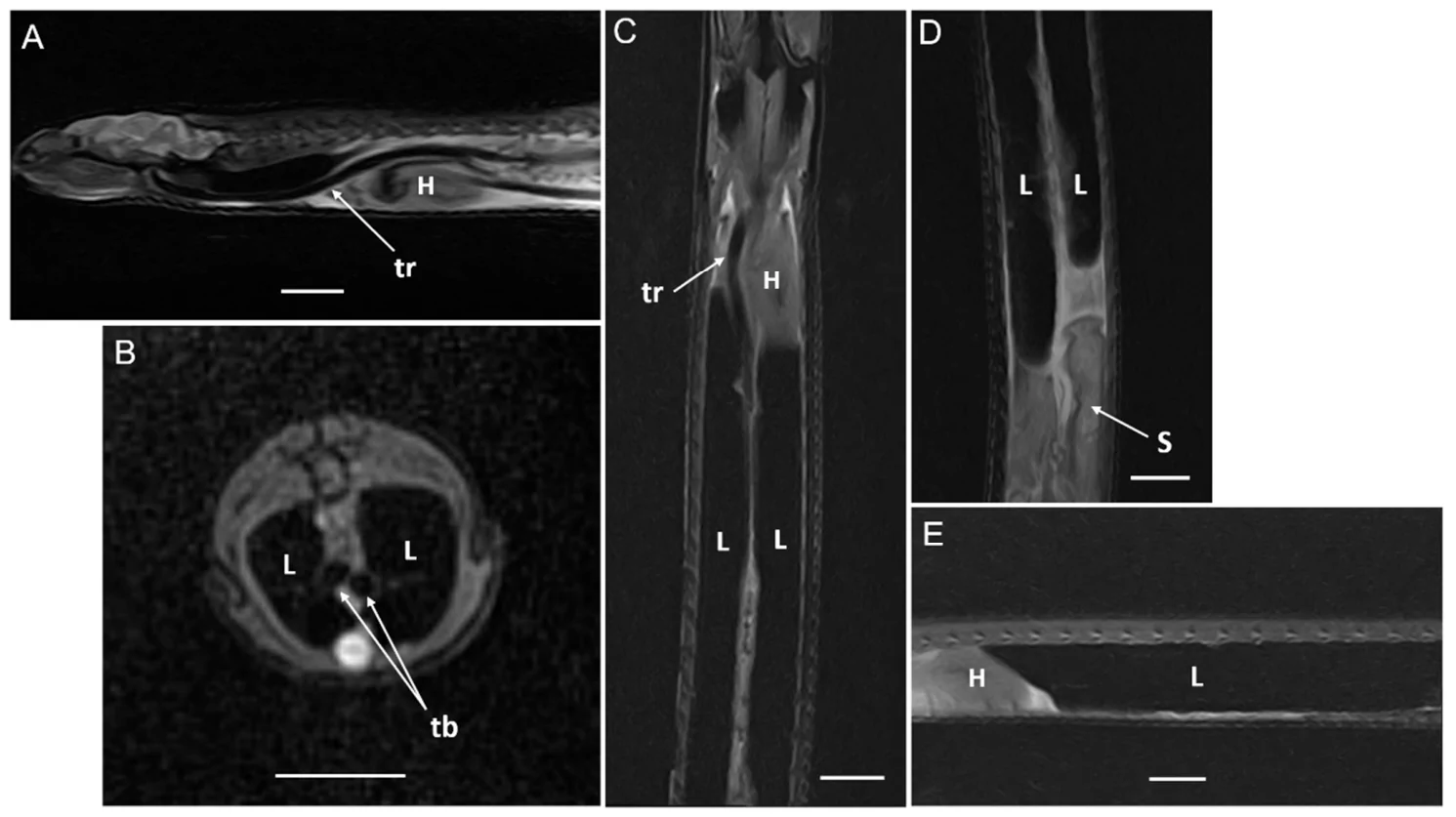
MRI images of a *Pseudopus apodus* in sagittal (**A**,**E**), transverse (**B**) and dorsal (**C**,**D**) planes obtained with a high-field system. Images (**A**,**C**–**E**) were acquired with a T2-weighted FSE 2D sequence and image (**B**) with a T1-weighted FSE 2D FatSAT sequence. Trachea, tracheal bifurcation, lungs, heart and stomach are identified. See the list for abbreviations. Bar = 10 mm.

**Figure 14:**
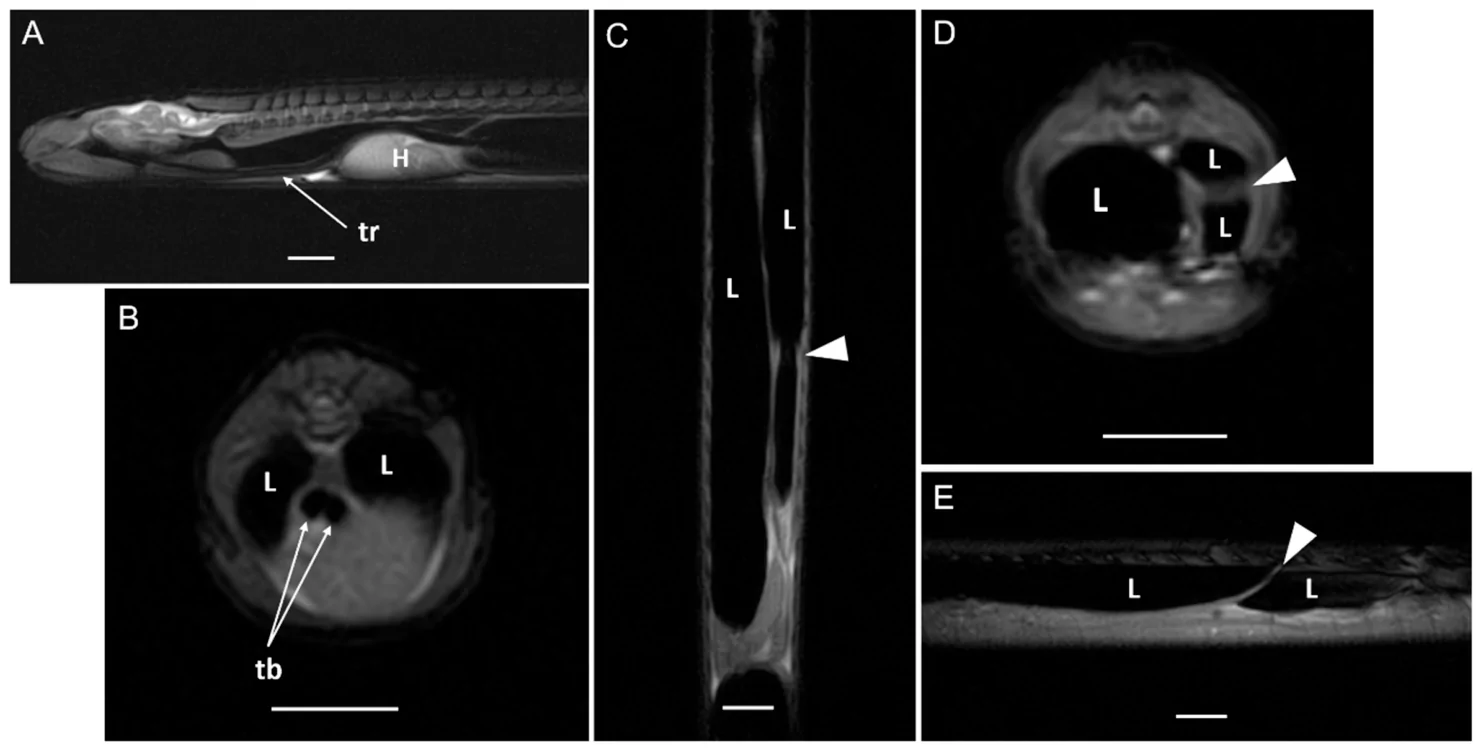
MRI images of a *Pseudopus apodus* in sagittal (**A**,**E**), transverse (**B**,**D**) and dorsal (**C**) planes obtained with a low-field system. They were acquired with a gradient-echo B-FFE 3D sequence. Trachea, tracheal bifurcation, lungs and heart are identified. See the list for abbreviations. A partial septation is seen in the caudal part of the left lung (arrowhead). Bar = 10 mm.

**Figure 15:**
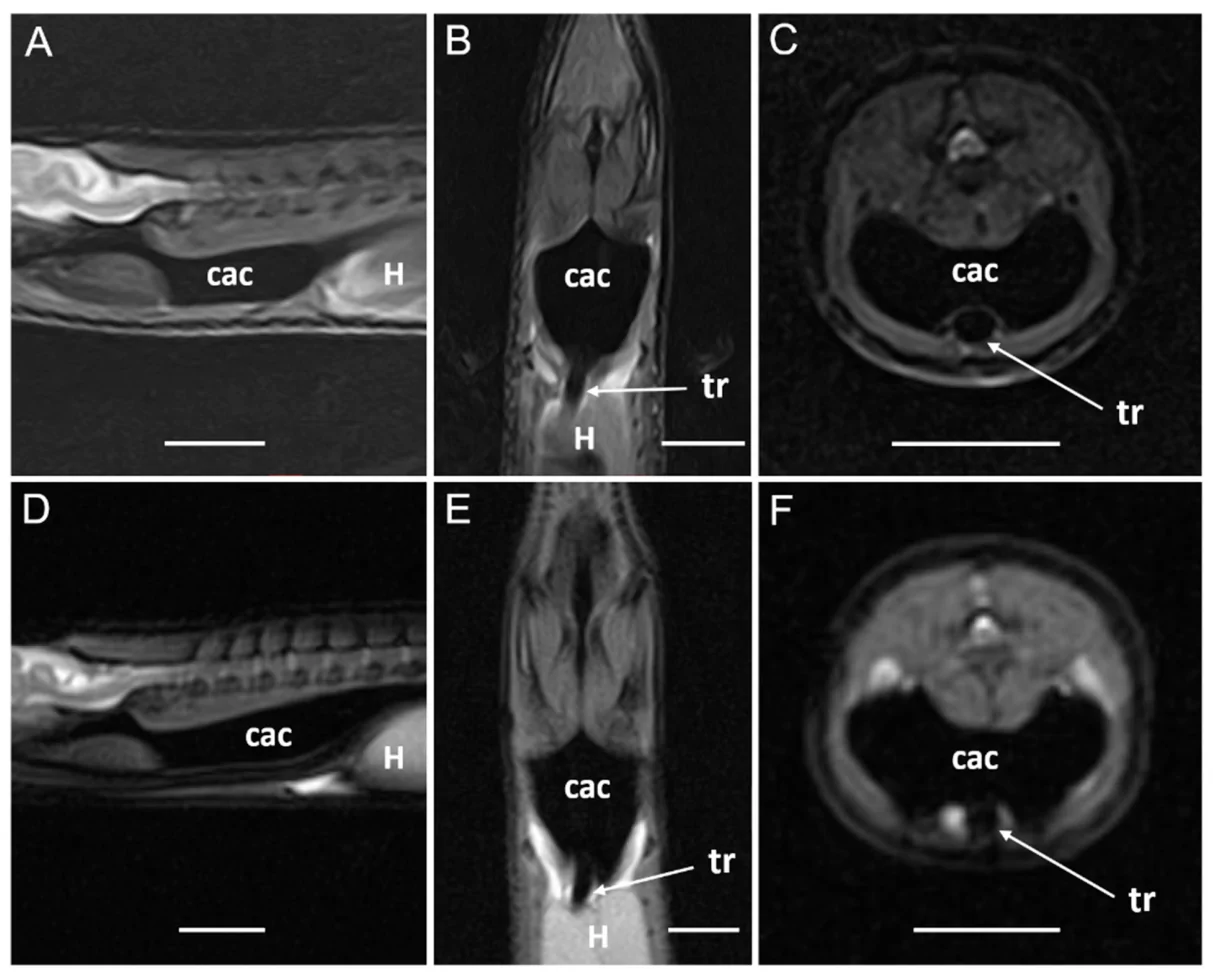
MRI images of a *Pseudopus apodus* in sagittal (**A**,**D**), dorsal (**B**,**E**) and transverse (**C**,**F**) planes obtained with high-field (**A**–**C**) and low-field (**D**–**F**) systems. A T2-weighted FSE 2D sequence was used for images obtained with the high-field system and a gradient-echo B-FFE 3D sequence was employed for the low-field acquisition. The cervical air chamber, trachea and heart are identified. See the list for abbreviations. Bar = 10 mm.

**Figure 16:**
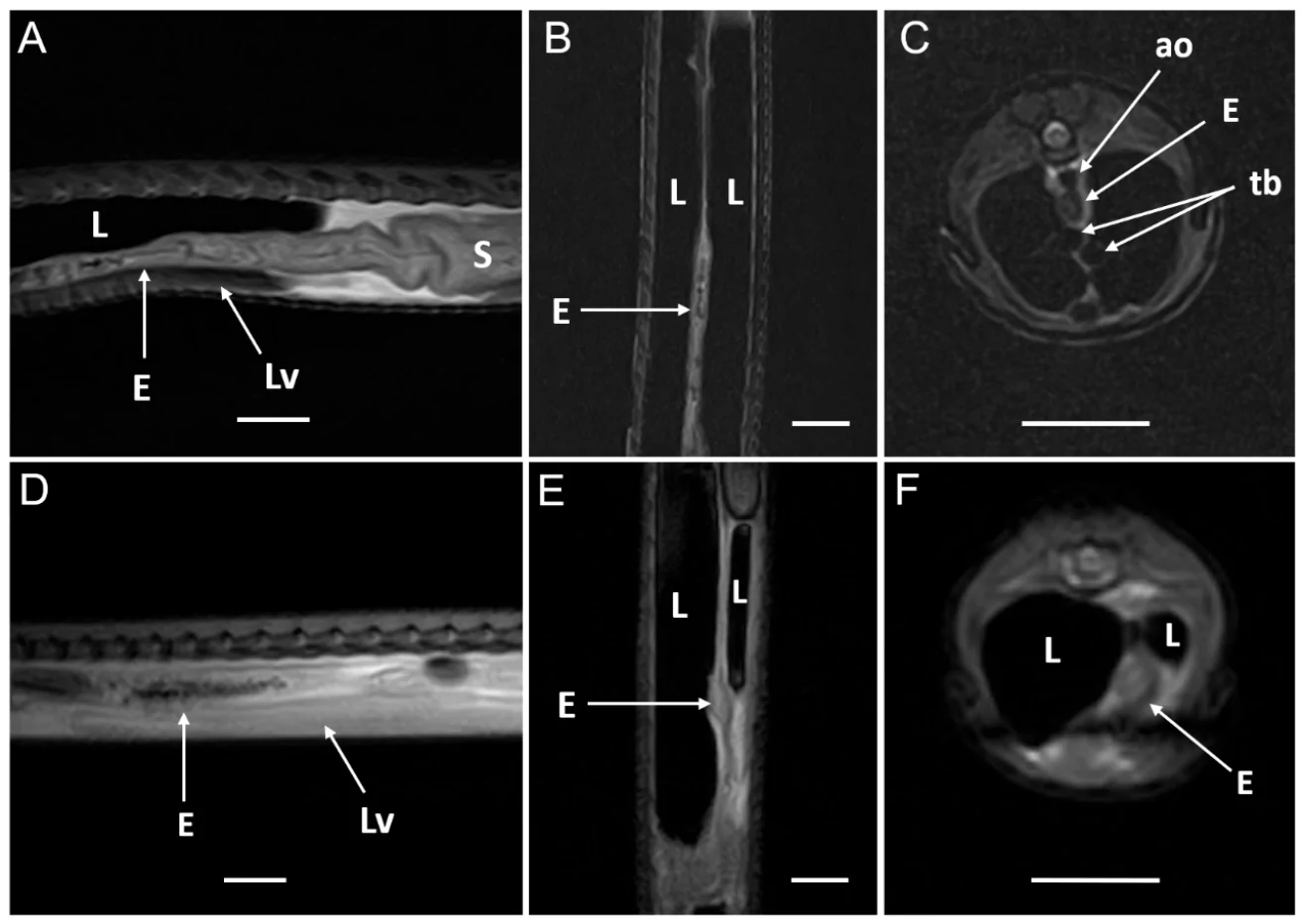
MRI images of a *Pseudopus apodus* in sagittal (**A**,**D**), dorsal (**B**,**E**) and transverse (**C**,**F**) planes obtained with high-field (**A**–**C**) and low-field (**D**–**F**) systems. A T2-weighted FSE 2D sequence was used for images obtained with the high-field system and a gradient-echo B-FFE 3D sequence was employed for the low-field acquisition. Esophagus, stomach, lung, liver, aorta and tracheal bifurcation are identified. See the list for abbreviations. Bar = 10 mm.

**Figure 17:**
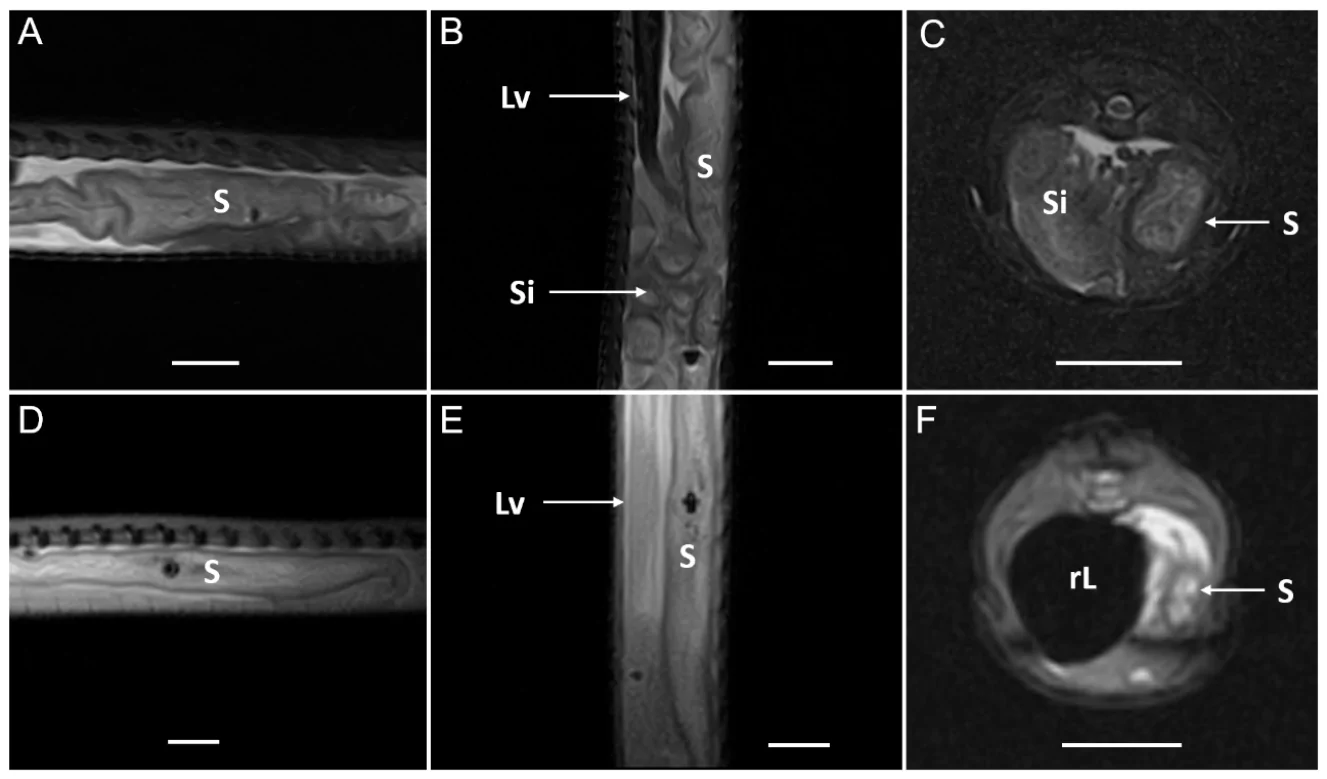
MRI images of a *Pseudopus apodus* in sagittal (**A**,**D**), dorsal (**B**,**E**) and transverse (**C**,**F**) planes obtained with high-field (**A**–**C**) and low-field (**D**–**F**) systems. A T2-weighted FSE 2D sequence was used for images obtained with the high-field system and a gradient-echo B-FFE 3D sequence was employed for the low-field acquisition. Stomach, liver, small intestine, and right lung are identified. See the list for abbreviations. Bar = 10 mm.

**Figure 18:**
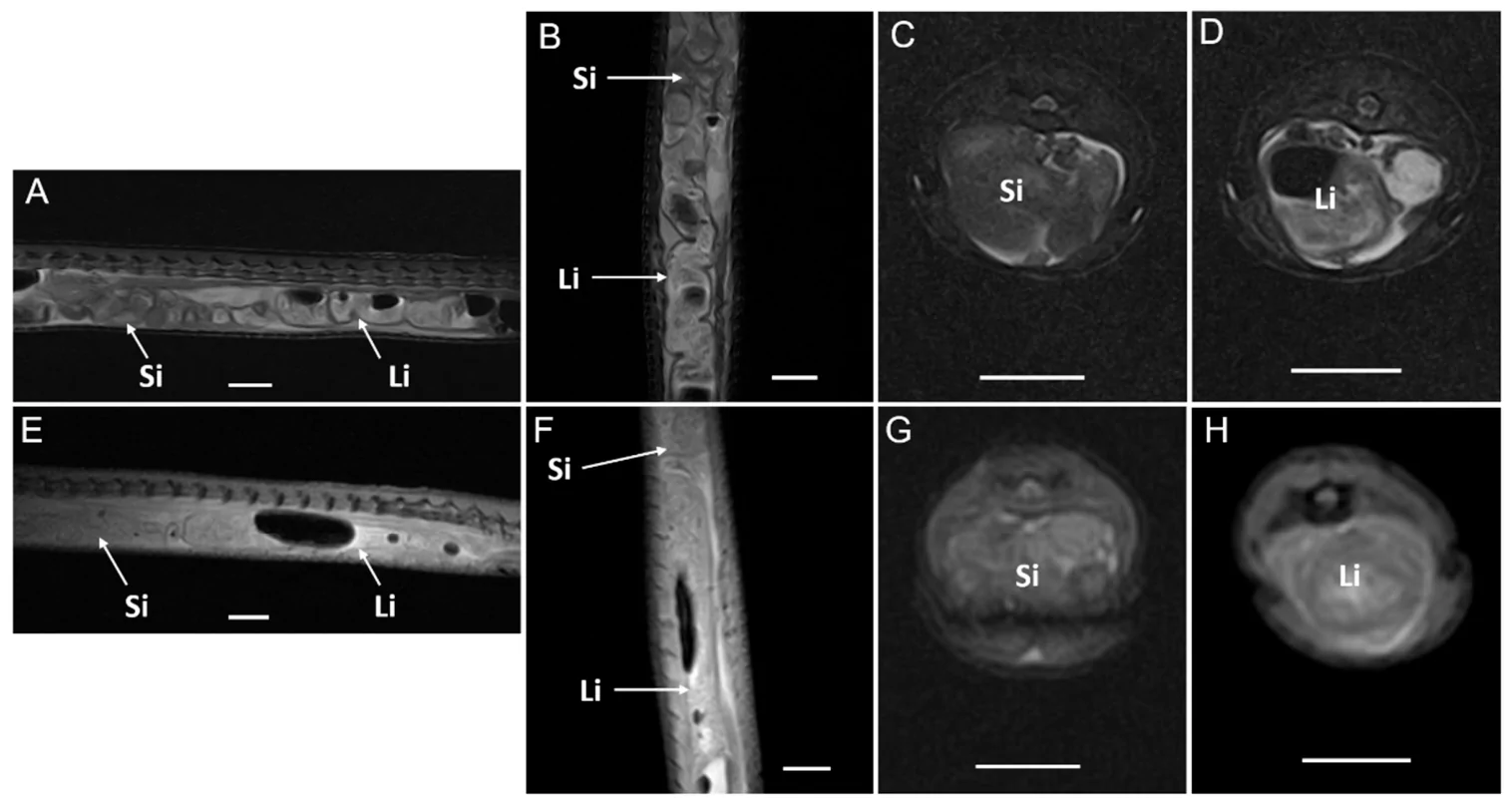
MRI images of a *Pseudopus apodus* in sagittal (**A**,**E**), dorsal (**B**,**F**) and transverse (**C**,**D**,**G**,**H**) planes obtained with high-field (**A**–**D**) and low-field (**E**–**H**) systems. A T2-weighted FSE 2D sequence was used for images obtained with the high-field system and a gradient-echo B-FFE 3D sequence was employed for the low-field acquisition. Small intestine and large intestine are identified. See the list for abbreviations. Bar = 10 mm.

**Figure 19:**
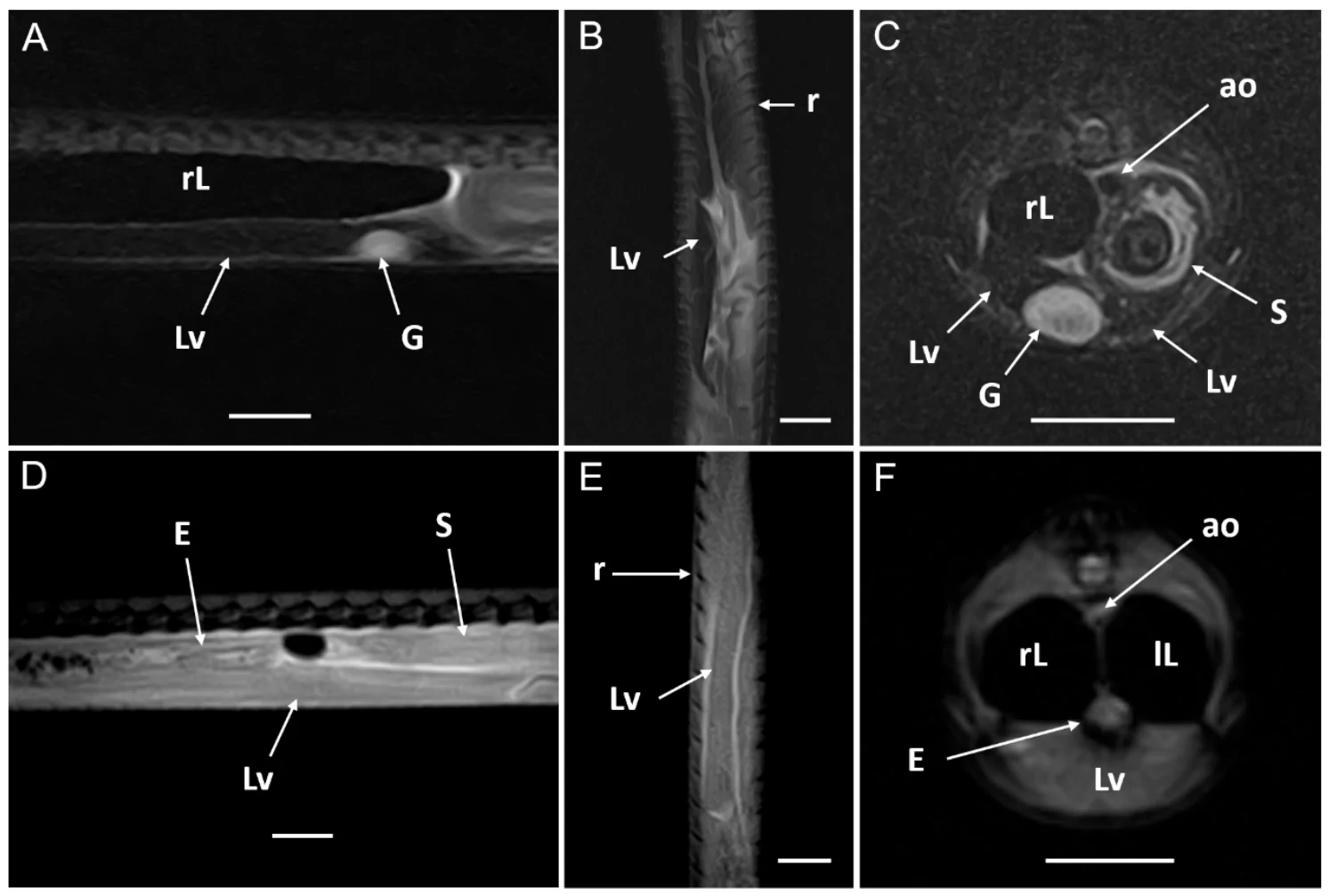
MRI images of a *Pseudopus apodus* in sagittal (**A**,**D**), dorsal (**B**,**E**) and transverse (**C**,**F**) planes obtained with high-field (**A**–**C**) and low-field (**D**–**F**) systems. A T2-weighted FSE 2D sequence was used for images obtained with the high-field system and a gradient-echo B-FFE 3D sequence was employed for the low-field acquisition. Liver, gallbladder, esophagus, stomach, right lung lobe, left lung lobe, aorta and ribs are identified. See the list for abbreviations. Bar = 10 mm.

**Figure 20:**
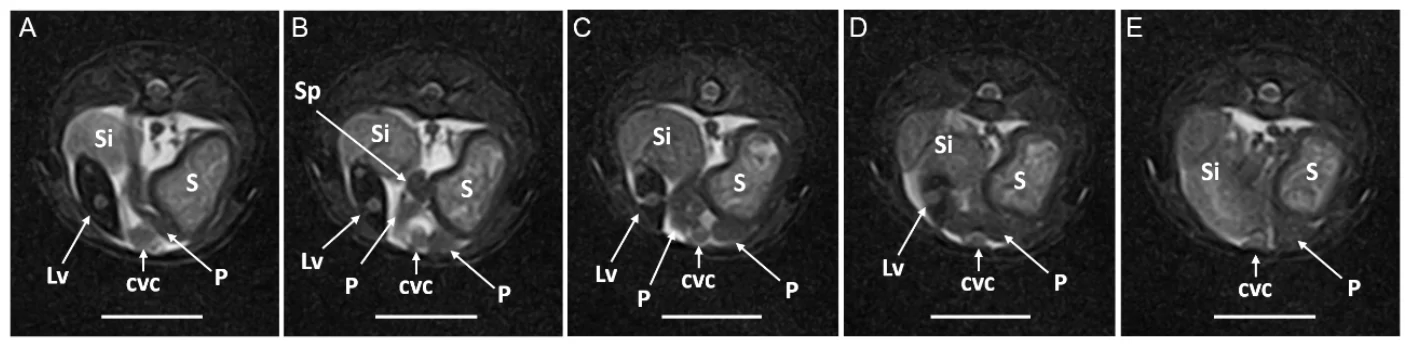
Contiguous MRI images of a *Pseudopus apodus* in transverse plane obtained with a high-field system (**A**–**E**). These images were acquired with a T2-weighted FSE 2D sequence. The series shows the complete pancreas, adjacent to the medial and ventral margin of the stomach. The spleen is visible in image (**B**). Liver, small intestine, stomach, caudal vena cava are also identified. See the list for abbreviations. Bar = 10 mm.

**Figure 21:**
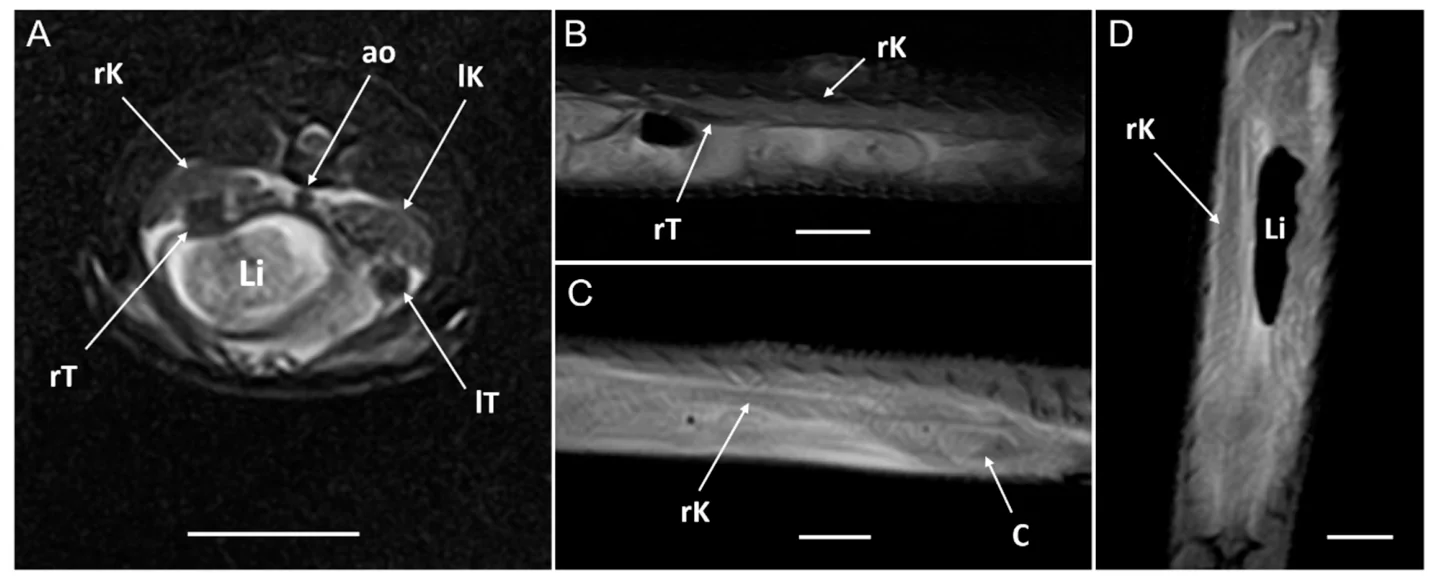
MRI images of a *Pseudopus apodus* in transverse (**A**) sagittal (**B**,**C**) and dorsal (**D**) planes obtained with high-field (**A**,**B**) and low-field (**C**,**D**) systems. A T2-weighted FSE 2D sequence was used for images obtained with the high-field system and a gradient-echo B-FFE 3D sequence was employed for the low-field acquisition. Right kidney, left kidney, right testis, left testis, large intestine, cloaca and aorta are identified. See the list for abbreviations. Bar = 10 mm.

**Figure 22:**
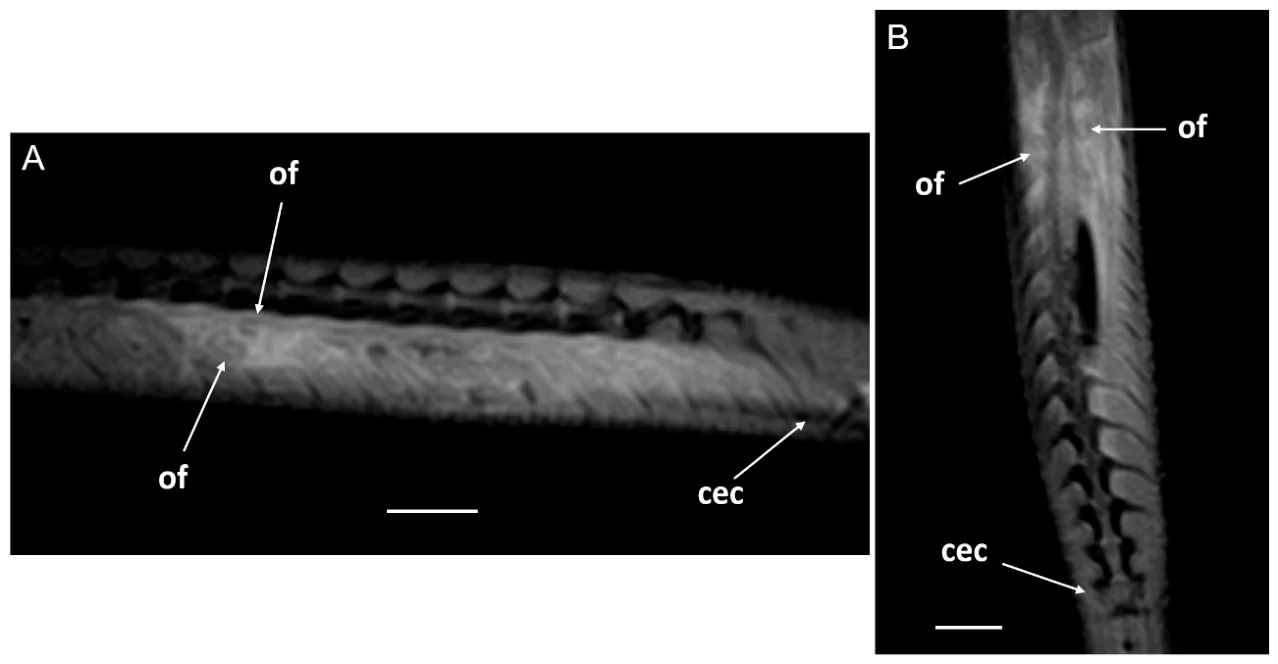
MRI images of a *Pseudopus apodus* in sagittal (**A**) and dorsal (**B**) planes obtained with a low-field system. These images were acquired with a gradient-echo B-FFE 3D sequence. Ovarian follicles and caudal end of the celomic cavity are identified. See the list for abbreviations. Bar = 10 mm.

**Figure 23:**
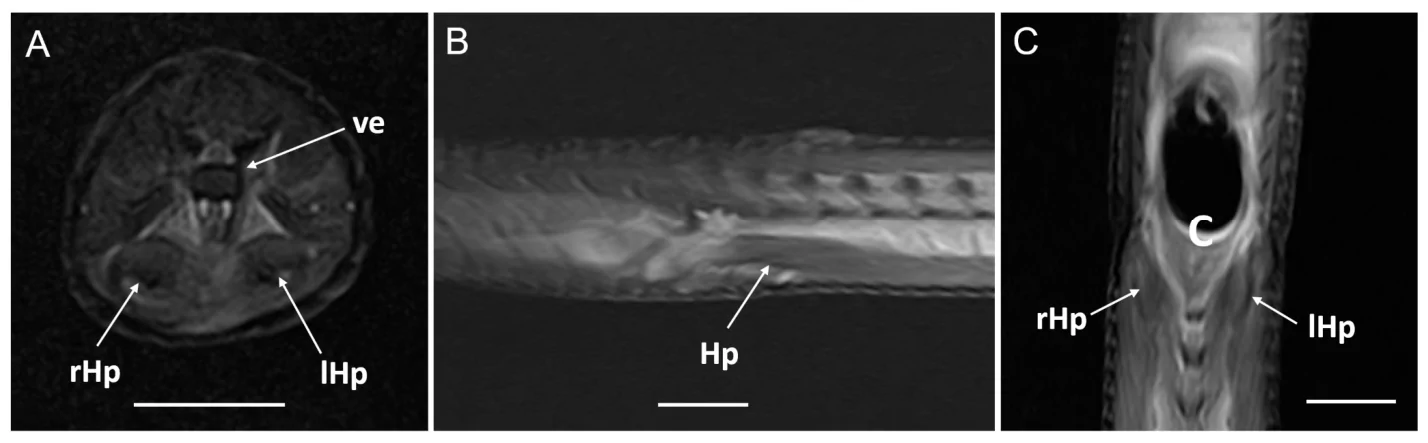
MRI images of a *Pseudopus apodus* in transverse (**A**), sagittal (**B**) and dorsal (**C**) planes obtained with a high-field system. These images were acquired with a T2-weighted FSE 2D sequence. Both hemipenes, vertebrae and cloaca are identified. See the list for abbreviations. Bar = 10 mm.

**Figure 24:**
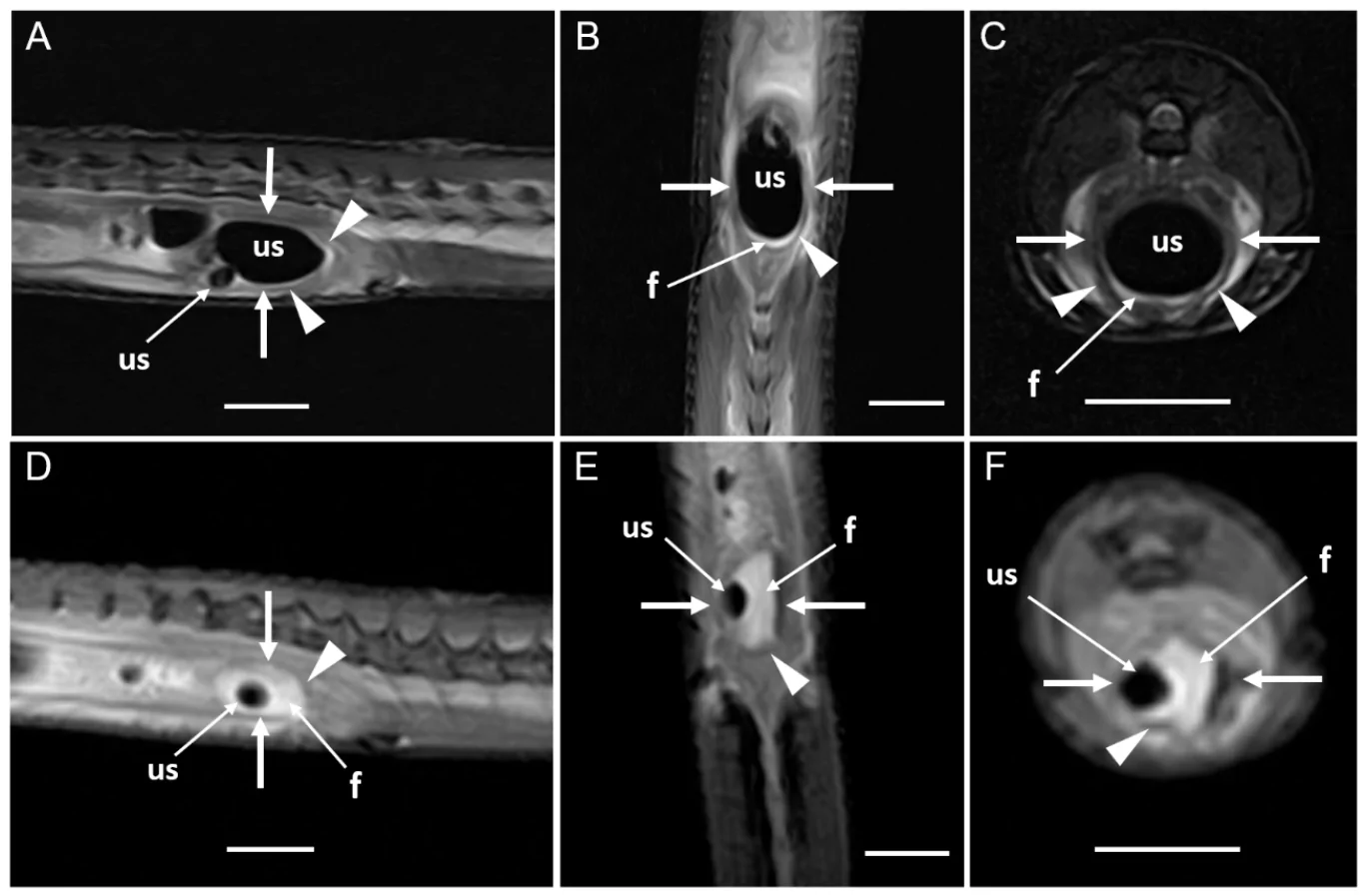
MRI images of a *Pseudopus apodus* in sagittal (**A**,**D**), dorsal (**B**,**E**) and transverse (**C**,**F**) planes obtained from a male specimen with a high-field (**A**–**C**) and from a female specimen with low-field (**D**–**F**) systems. A T2-weighted FSE 2D sequence was used for images obtained with the high-field system and a gradient-echo B-FFE 3D sequence was employed for the low-field acquisition. Cloaca is identified (between thick arrows). Wall of the cloaca appears as a fine hypointense line (arrowheads) delimiting hyperintense content (fluid) and hypointense intraluminal structures (urate salts). See the list for abbreviations. Bar = 10 mm.

**Table 1:** Main MRI acquisition parameters. See the list for abbreviations.

Magnetic Field	Sequence	Plane	TR (ms)	TE (ms)	NEX	FOV		SLTH (mm)	Matrix		#SL
						mm	mm		Pixel	Pixel	
High	FSE 2D T1WI FatSat	Transverse	533	10	1	90	103	2	224	256	38
High	FSE 2D T2WI	Transverse	5815–11,044	84	2–3	60–70	60–70	2	384–392	284–384	30–57
High	FSE 2D T2WI	Dorsal	1551–1939	84	4–5	120–135	150–170	2.5	704–832	836–996	8–10
High	FSE 2D T2WI	Sagittal	1939–2908	84	3–4	130–150	160–190	2–2.5	704–768	836–996	9–15
High	FSE 2D T2WI	Whole body sagittal	1939	84	3	150	400	2	640	1708	10
Low	B-FFE 3D	Transverse	10–17	5–7	2–4	160–200	160–200	2	432–450	432–450	18–56
Low	B-FFE 3D	Dorsal	14	7	2	160–180	160–180	2	432	432	12
Low	B-FFE 3D	Sagittal	14	7	2–4	135–200	160–200	2	366–432	366–432	8–16

**Table 2:** Anatomical structures identified and not identified using the low-field and the high-field.

Anatomic Structure	Low-Field MRI System	High-Field MRI System
Eyes	Yes	Yes
Third eyelid	Yes	Yes
Brain	Yes	Yes
Tongue	Yes	Yes
Pterygoid muscles	Yes	Yes
Axial muscles	Yes	Yes
Glottis	Yes	Yes
Cervical air chamber	Yes	Yes
Trachea	Yes	Yes
Atria	Yes	Yes
Ventricle	Yes	Yes
Sinus venosus	Yes	Yes
Tracheal bifurcation	Yes	Yes
Lungs	Yes	Yes
Esophagus	Yes	Yes
Stomach	Yes	Yes
Small intestine	Yes	Yes
Large intestine	Yes	Yes
Liver	Yes	Yes
Gallbladder	Yes	Yes
Kidneys	Yes	Yes
Cloaca	Yes	Yes
Otic clefts	Yes	Yes
Opening of the vomeronasal organ—choanae	Yes	Yes
Osteoderms	Yes	Yes
Mandible	Yes	Yes
Nasal cavity ethmoturbinates	Yes	Yes
Teeth	Yes	Yes
Vertebrae	Yes	Yes
Ribs	Yes	Yes
Hyoid bone (partially)	Yes	Yes
Spinal cord	No	Yes
Thyroid gland	No	Yes
Cranial cava veins	No	Yes
Aortic arches	No	Yes
Dorsal aorta	Yes	Yes
Caudal vena cava	No	Yes
Iliac veins bifurcation	No	Yes
Dorsal vein of the tail	No	Yes
Spleen	No	Yes
Pancreas	No	Yes
Testes	No	Yes
Hemipenes	No	Yes
Optic nerves	Yes	No
Ovarian follicles	Yes	Not evaluated

## Data Availability

The authors confirm that the data supporting the findings of this study are available within the article.

## Abbreviations

2D	two-dimensional
3D	three-dimensional
a	atria
aa	aortic arches
Ag	adrenal glands
am	axial muscles
ao	aorta dorsal
B	bladder
br	brain
B-FEE	balanced fast field echo
C	cloaca
cac	cervical air chamber
Cb	cerebellum
cec	caudal end of celomic cavity
CT	computed tomography
cv	cranial cava veins
cvc	caudal vena cava
Cx	cortex
dv	dorsal vein of the tail
E	esophagus
el	eyelid
ey	eye
f	fluid
FatSAT	fat-saturated
FFE	fast field echo
FOV	field of view
FSE	fast spin-echo
G	gallbladder
gt	glottis
H	heart
Hp	hemipenis
hy	hyoid bone
iv	iliac veins bifurcation
IVv	fourth ventricle
Kd	kidney
lg	lateral groove
L	lung
lHp	left hemipenis
Li	large intestine
lK	left kidney
lL	left lung
lT	left testis
Lv	liver
m	mandible
MRI	magnetic resonance imaging
nc	nasal cavity
NEX	number of averaged experiments
OB	olfactory bulb
oc	otic cleft
od	osteoderms
of	ovarian follicles
on	optic nerve
OT	optic tectum
P	pancreas
pm	pterygoideus muscle
r	rib
Rhom	rhombencephalon
rHp	right hemipenis
rK	right kidney
rL	right lung
rT	right testis
S	stomach
sc	spinal cord
Si	small intestine
SLTH	slice thickness
Sp	spleen
sv	sinus venosus
T1WI	T1-weighted
T2WI	T2-weighted
t	teeth
tb	tracheal bifurcation
TE	echo time
Th	thyroid gland
to	tongue
tr	trachea
TR	repetition time
us	urate salts
v	ventricle
ve	vertebrae
vn	opening of the vomeronasal organ and choanae
#SL	number of slices
